# Induction of Lysosome Membrane Permeabilization as a Therapeutic Strategy to Target Pancreatic Cancer Stem Cells

**DOI:** 10.3390/cancers12071790

**Published:** 2020-07-04

**Authors:** Timothy P. Cash, Sonia Alcalá, María del Rosario Rico-Ferreira, Elena Hernández-Encinas, Jennifer García, María Isabel Albarrán, Sandra Valle, Javier Muñoz, Sonia Martínez-González, Carmen Blanco-Aparicio, Joaquín Pastor, Manuel Serrano, Bruno Sainz

**Affiliations:** 1 Tumor Suppression Group, Spanish National Cancer Research Centre (CNIO), 28029 Madrid, Spain; tim.cash@senolytx.com (T.P.C.); manuel.serrano@irbbarcelona.org (M.S.); 2 Department of Cancer Biology, Instituto de Investigaciones Biomédicas “Alberto Sols” (IIBM), 28029 Madrid, Spain; sonia.alcala@uam.es (S.A.); san.valle@predoc.uam.es (S.V.); 3 Department of Biochemistry, Universidad Autónoma de Madrid (UAM), 28029 Madrid, Spain; 4 Chronic Diseases and Cancer, Area 3—Instituto Ramón y Cajal de Investigación Sanitaria (IRYCIS), 28034 Madrid, Spain; 5 Experimental Therapeutics Programme, Spanish National Cancer Research Centre (CNIO), 28029 Madrid, Spain; ricoferreiram@gmail.com (M.d.R.R.-F.); mehernandez@cnio.es (E.H.-E.); jgarocha@externas.sescam.jccm.es (J.G.); mialbarran@cnio.es (M.I.A.); smartinezg@cnio.es (S.M.-G.); cblanco@cnio.es (C.B.-A.); jpastor@cnio.es (J.P.); 6 Proteomics Unit–ProteoRed-Instituto de Salud Carlos III, Spanish National Cancer Research Centre (CNIO), 28029 Madrid, Spain; jmunozpe@cnio.es; 7 Institute for Research in Biomedicine (IRB Barcelona), The Barcelona Institute of Science and Technology (BIST), 08028 Barcelona, Spain; 8 Catalan Institution for Research and Advanced Studies (ICREA), 08028 Barcelona, Spain

**Keywords:** compound library, cancer stem cells, pancreatic ductal adenocarcinoma, lysosomal membrane permeabilization, patient-derived xenografts

## Abstract

Despite significant efforts to improve pancreatic ductal adenocarcinoma (PDAC) clinical outcomes, overall survival remains dismal. The poor response to current therapies is partly due to the existence of pancreatic cancer stem cells (PaCSCs), which are efficient drivers of PDAC tumorigenesis, metastasis and relapse. To find new therapeutic agents that could efficiently kill PaCSCs, we screened a chemical library of 680 compounds for candidate small molecules with anti-CSC activity, and identified two compounds of a specific chemical series with potent activity in vitro and in vivo against patient-derived xenograft (PDX) cultures. The anti-CSC mechanism of action of this specific chemical series was found to rely on induction of lysosomal membrane permeabilization (LMP), which is likely associated with the increased lysosomal mass observed in PaCSCs. Using the well characterized LMP-inducer siramesine as a tool molecule, we show elimination of the PaCSC population in mice implanted with tumors from two PDX models. Collectively, our approach identified lysosomal disruption as a promising anti-CSC therapeutic strategy for PDAC.

## 1. Introduction

It is now generally accepted that the majority of solid tumors contain a small sub-population of highly plastic, tumorigenic and chemoresistant cells, known as cancer stem cells (CSCs) or cancer-initiating cells, which are the drivers of tumor evolution, metastasis and tumor relapse [1,2]. Thus, from a clinical perspective, targeting and eliminating the CSC compartment would ensure a more effective tumor eradication [2,3,4,5]; however, to date, only a handful of therapeutics specifically targeting CSCs have advanced to clinical trials (reviewed in Saygin et al. [6]). While direct, cell-based chemical screenings of CSCs have the potential to detect new anti-CSC inhibitors, such platforms are plagued by numerous technical and biological limitations [7]. For example, the use of markers expressed on the cell surface of CSCs, side-population or intracellular enzymatic activities (e.g., ALDH1 activity) have been used to isolate and enrich for CSCs, but these methods are limited due to 1) the lack of clearly defined CSC-specific markers 2) CSC phenotypic heterogeneity, 3) the propensity of enriched CSCs to undergo asymmetric division to restore the original cellular distribution of CSCs and non-CSCs prior to enrichment (i.e., CSC plasticity), and 4) the technical limitation of isolating large numbers of highly purified CSC populations. Thus, long-term cultures of purified CSCs isolated based on the expression of cell surface markers or enzymatic activity are technically unsustainable and not amenable to compound screening. Alternative phenotypic/functional approaches based on methodologies such as in vitro sphere cultures have also been utilized, but such approaches are labor-intensive, the heterogeneity of the cultures cannot be controlled for, and spheres are inherently difficult to manipulate making this approach very low-throughput.

We took an alternative approach and describe in this study a small-scale screen using mouse embryonic stem cells (mESCs) as a surrogate system for CSCs to identify small-molecules with anti- “stem” properties. Unlike CSCs, mESCs are readily and homogeneously propagated making them amenable to screening, but at the same time share relevant key properties with CSCs in that they 1) are highly plastic and give rise to diverse cellular lineages 2) express markers of pluripotency (e.g., Klf4, Oct3/4, Sox2 and Nanog) and 3) have tumorigenic potential. Thus, mESCs, despite being obviously different from CSCs, represent a suitable surrogate model for identifying inhibitors of CSCs properties, including self-renewal, the latter of which is governed by transcriptional subprograms described to be similar to that of ESCs [8,9]. 

Using the aforementioned approach, we identified a class of molecules with preferential CSC killing activity. In characterizing the mechanism of action of these molecules, we identified a general strategy to target CSCs: lysosomal membrane permeabilization (LMP) activity, a well-described mechanism of action for a wide range of chemotherapeutics. LMP causes the release of lysosomal contents (e.g., cathepsins, proteases and other hydrolases) to the cytosol, a potentially lethal process as released lysosomal proteases—primarily cathepsins—can affect important cellular organelles (e.g., mitochondria) and degrade essential proteins causing lysosomal-dependent cell death (LDCD) [10,11]. Several reports have documented changes in lysosomes during cancer progression and metastasis, including changes in lysosomal size and volume [12], increased membrane fragility [13], trafficking [14] and composition [15]. Moreover, a correlation has been shown between the metastatic capacity and aggressiveness of tumor cells and high lysosomal cysteine cathepsins activity, such that these cells are especially sensitive to LDCD [16]. Thus, there has been much interest in understanding lysosomes and lysosome-associated proteins as potential therapeutic targets in cancer, and as such, therapies targeting tumor lysosomes to induce LDCD are under development [17]. Indeed, several classes of LMP-inducing drugs that preferentially target cancer cell lysosomes have been described [18,19]. Moreover, there is evidence indicating that glioblastoma and leukemic CSCs present an enhanced susceptibility to LMP-inducing agents [19,20]. Herein we have identified and characterized a new LMP-inducing molecule that shows potent anti-cancer activity. Specifically, we show that the specificity of LMP-inducing agents, in general, can reach beyond that of just cancer cells, as we show that CSCs are more sensitive to LMP drugs than their non-CSC counterparts and these, in turn, are more sensitive than normal cells. Thus, the lysosome can be considered a new Achilles’ heel of CSCs, and the compounds described in this study are attractive candidates to be further developed for clinical use.

## 2. Results

### 2.1. Identification of Novel Compounds That Compromise CSC Viability

**Figure 1:**
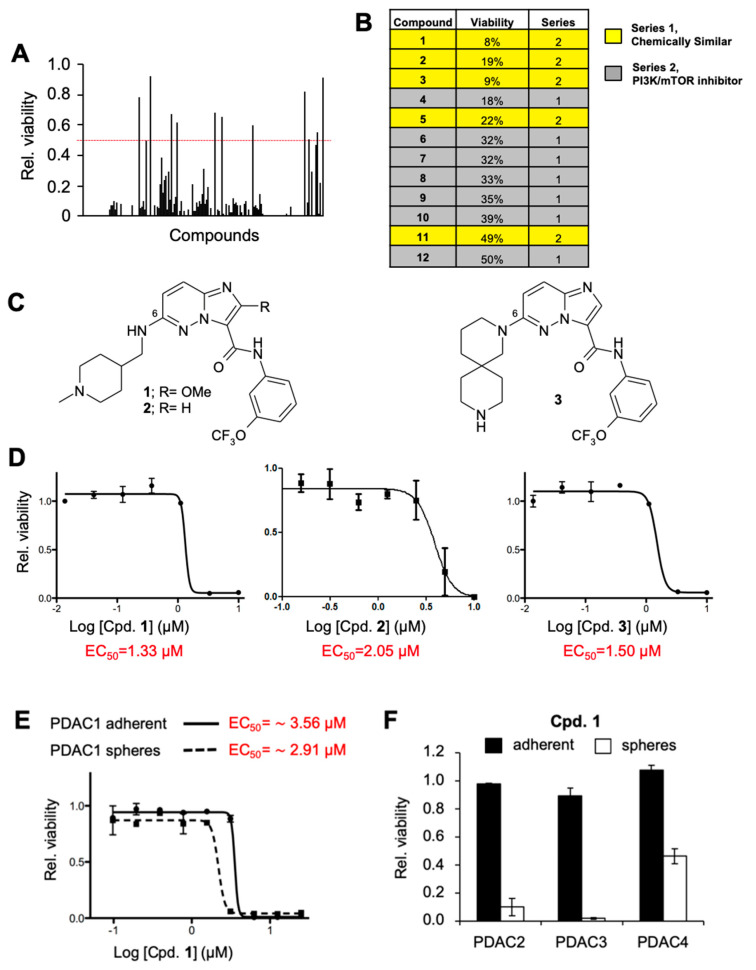
Identification of novel compounds that compromise cancer stem cell (CSC) viability. (**A**) Analysis of relative (rel.) cell viability for mouse embryonic stem cells (mESCs) following 30 h treatment with 5 µM of each of the 640 compounds contained within the chemical library. Red line indicates 50% viability cutoff. (**B**) Top 12 compounds that increased the percentage of non-viable cells by at least 50% and the series to which they belonged. (**C**) Chemical structure of the structurally similar series of a -imidazo[1,2-b]pyridazine-3-carboxylic acid (3-trifluoromethoxy-phenyl)-amide derivatives with substitution at the 6-position with several “amino-fragments”. (**D**) Viability curves performed on mESCs treated with increasing concentrations of compounds (Cpd.) 1, 2 and 3 for 30 h. Indicated in red are the calculated half maximal effective concentration (EC_50_). (**E**) Viability curves performed on PDAC1 adherent 2D monolayers or 3D spheres treated with increasing concentrations of compound Cpd. 1 for 30 h. Indicated in red are the calculated EC_50_. (**F**) Analysis of rel. cell viability ± standard deviation for PDAC2, PDAC3 and PDAC4 adherent 2D monolayers or 3D spheres following 30 h treatment with 2.5 µM of Cpd. 1.

We screened a chemical library of 640 compounds designed to represent a diverse chemical space (see Materials and Methods). Following 30-hour treatment with 5 µM of each compound, we assessed mESCs viability by a standard MTS assay and identified 12 compounds that decreased the number of viable cells by at least 50%, belonging to 2 classes (Figure 1A,B). One class (Series 2) comprised seven compounds previously identified to directly inhibit PI3K and/or mammalian target of rapamycin (mTOR) kinase activity in vitro, and which we revalidated herein (Figure S1A). The role of the PI3K/mTOR pathway in both mESC and CSC biology is well described [21,22,23,24,25,26] and, therefore, the identification of these compounds validates the effectiveness of the screen. The second class (Series 1) did not show activity against PI3K/mTOR in vitro (Figure S1A) and were structurally similar imidazo [1,2-b]pyridazine-3-carboxylic acid (3-trifluoromethoxy-phenyl) amide derivatives with the 6-position substituted with different “amino-fragments” (Figure 1C). 

To validate and further prioritize the hits from Series 1, we generated viability dose response curves in mESCs for the top three hits. Consistent with the relative activities determined from the initial screening, compounds (Cpd) 1–3, showed significant activity against mESCs viability with half maximal effective concentrations (EC_50′_s) of 1.33 µM, 2.05 µM and 1.50 µM, respectively (Figure 1D). Next, we evaluated the effects of Cpd 1 and 3 in human CSCs by first assessing their cytotoxicity on the PDX-derived PDAC culture PDAC1. These cells were grown in adherence or as spheres, where the latter enriches for the CSC population in vitro. While both Cpds 1 and 3 decreased the viability of PDAC1 cells, the effect was more pronounced on CSC-enriched spheres compared to cells grown as adherent monolayers (Figure 1E and Figure S1B). Since Cpd 1 showed the most potent activity, it was selected for follow-up assays on a panel of PDX-derived cultures. Confirming activity across multiple primary cultures, Cpd 1 at a 2.5 µM dose showed similar and preferential anti-CSC activity against three additional PDAC cell lines when cultured as spheres vs. adherent monolayers (PDAC 2–4) (Figure 1F). To rule out the possibility that the effects observed were due to culture conditions (2D adherent versus 3D spheres), we treated 7-day-old spheres re-plated as adherent 2D cultures with Cpd 1 for only 24 h so that they retain their CSC properties (Figure S1C). As expected, the higher activity of the compound for CSCs was independent of the type of culture condition (Figure S1D).

### 2.2. Compound 1 Impairs the Self-Renewal and Tumor Initiating Capacity of PDAC Cells by Depleting the CSC Compartment 

**Figure 2:**
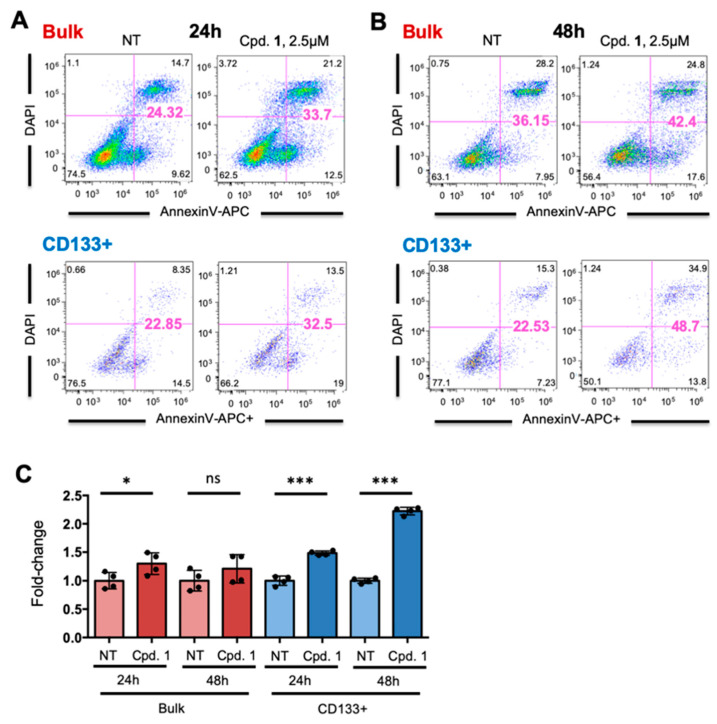
Compound 1 preferentially targets CD133+ CSCs. (**A**,**B**) Representative flow cytometry plots of the percentage of early apoptosis (Annexin-V+), late apoptosis (Annexin-V+/DAPI+) and dead (DAPI+) cells in the total PDAC2 cell population (top) or within the CD133-positive population (bottom) treated with Cpd. 1 at 2.5 µM. Cells were analyzed (**A**) 24 h and (**B**) 48 h post treatment initiation. (**C**) Fold-change in total apoptosis (early and late apoptosis) for each group and treatment shown in panels A and B, (Student’s t-test). * *p* < 0.05; *** *p* <0.001; ns, not significant.

Having observed a preferential cytotoxic effect on human PDAC CSCs, we next asked whether Cpd 1 affects key CSCs properties and biological functions. First, we confirmed by flow cytometry that Cpd 1 preferentially targets the CSC compartment by analyzing the percentage of bulk and CD133+ cells undergoing apoptosis 24 and 48 h post treatment (Figure 2A–C). Indeed, when CD133+ cells were analyzed separately, we observed that Cpd 1 induced significantly more apoptosis in the PDAC2 CD133+ population compared to the bulk population 24 and 48 h post treatment (Figure 2C). 

**Figure 3:**
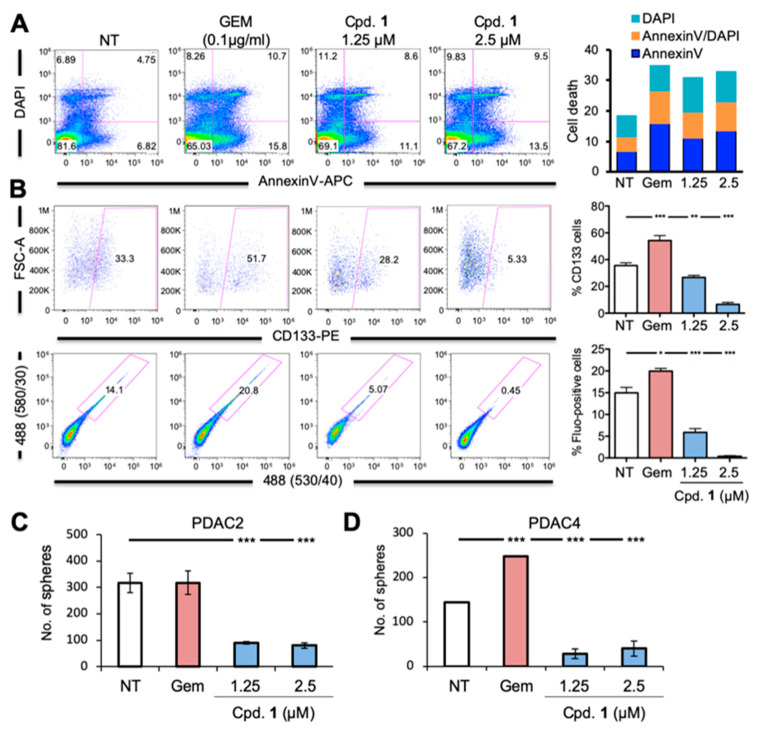

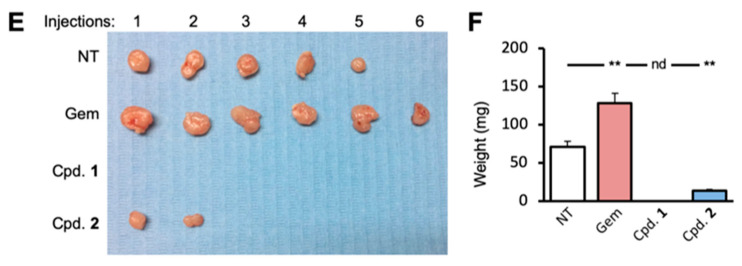
Series 1 compounds impair self-renewal and tumor initiating capacity of PDAC cells by depleting the CSC compartment. (**A**) Left: Representative flow cytometry plots of the percentage of early apoptosis (Annexin-V+), late apoptosis (Annexin-V+/DAPI+) and dead (DAPI+) cells in PDAC2 cells treated with 0.1 µg/mL gemcitabine (GEM) or Cpd. 1 at 1.25 or 2.5 µM. Apoptosis was determined 72 h post treatment initiation. Right: Sum of early apoptosis, late apoptosis and dead cell percentages for each treatment. (**B**) Left: Representative flow cytometry plots of CD133-PE or autofluorescence (Fluo) expression (percentage) in PDAC4 cells 72 h following treatment with 0.1 µg/mL GEM or Cpd. 1 at 1.25 or 2.5 µM. Right: Percentage (%) of CD133 or Autofluorescent-positive cells ± standard deviation for each treatment. (*n* = 4–6 per sample; Student’s t-test). (**C–D**) Number of 1st generation spheres/mL ± standard deviation for (**C**) PDAC2 or (D) PDAC4 cultures treated for with 0.1 µg/mL GEM or Cpd. 1 at 1.25 or 2.5 µM (*n* = 6; Student’s t-test). (**E**) Images of tumors obtained 10 weeks after injection of 5 × 10^3^ PDAC4 spheres treated for 3 days with 0.1 µg/mL GEM or Cpd. 1 or Cpd. 2 at 1.25 µM (*n* = 6 injections per treatment). (**F**) Summary of weights of tumors shown in (E), (Student’s *t*-test). *p < 0.05; **p < 0.01; ***p <0.001; nd, not determined.

We next compared the decline in CD133-positive or autofluorescent-positive [27] CSC populations in cultures treated with Cpd 1 or gemcitabine, the standard of care chemotherapeutic for PDAC and to which CSCs are inherently resistant [28]. Adherent cultures of PDAC2 or PDAC4 were treated for 3 days with gemcitabine or Cpd 1 at 2.5 µM or 1.25 µM (Figure 3A–D). We first assessed the capacity of each treatment to induce apoptosis (Figure 3A) and modulate the percentage of CD133-positive or autofluorescent-positive CSCs (Figure 3B) in PDAC4 cells by flow cytometric analysis. While all three treatments induced apoptosis, treatment with gemcitabine preferentially targeted the non-CSC (CD133-negative or autofluorescent-negative) population evident by the increase in the percentage CD133-positive or autofluorescent-positive cells following treatment. In contrast, treatment with both doses of Cpd 1 led to both an increase in apoptosis and a significant decrease in the percentage of CD133-positive or autofluorescent-positive cells, indicating again selective elimination of PDAC CSCs (Figure 3A,B). We then assessed the effect of gemcitabine or Cpd 1 on self-renewal by assessing sphere-forming capacity and, as expected, found that while gemcitabine either did not affect sphere formation in the case of PDAC2 or increased it in the case of PDAC4 (Figure 3C,D), Cpd 1 decreased sphere forming capacity at both doses tested and in both PDAC lines (Figure 3C,D). Finally, in order to determine if Cpd 1 impacts tumor initiating capacity, we treated sphere-derived PDAC2 cells for 3 days with either gemcitabine or Cpd 1 at 1.25 µM, and subsequently subcutaneously injected non-treated and treated cells in vivo. Four weeks post injection we observed that while cells pre-treated with gemcitabine gave rise to larger tumors, Cpd 1-treated cells exhibited a complete inhibition in tumor formation (Figure 3E,F). This reduced tumor-initiating capacity correlates with the inhibitory effects of Cpd 1 on CSCs seen in vitro (Figure 3A–D).

### 2.3. Characterization and Development of Chemical Probes to Study the Mechanism of Action of Series 1 Compounds

In order to more fully understand the mechanism by which Series 1 hits impact CSC viability, self-renewal and tumor initiating capacity, we first carried out a structure-activity relationship (SAR) analysis, comparing the activity of Cpd 1 against a series of structural analogs (Figure S2) including hit Cpd 2. After performing dose-response viability assays on PDAC1 spheres, we observed that the imidazo[1,2-b]pyridazine scaffold of Cpds 1 and 2 could be replaced by imidazo[1,2-a]pyrazine bicycle (Cpd 17). Importantly, we observed that the activity of this class of compounds depended on the presence of a basic nitrogen residue at C-6 position of the imidazo[1,2-b]pyridazine moiety as revealed by the lack of activity of Cpds 19 and 21. Noteworthily, all compounds in this series with a basic profile (pKa > 7) retained activity with EC_50′_s below 10 µM whereas non-basic compounds (pKa < 7) had no activity, even at 50 µM (Figure S2). Thus, as an example, Cpd 16 bearing a piperidine secondary amine moiety, showed good potency (EC_50_ = 3.26 µM) but its corresponding analog Cpd 15, where the basicity was removed by the protection of the N-atom with a tert-butyl carbamate group, completely lost activity. The non-basic Cpds 13 and 18 with a hydroxyl group in the 6-position fragment instead of a basic amine were also inactive. Similar behavior was observed for Cpd 19 substituted with a non-basic tetrahydropyranyl ring. Furthermore, the non-basic cyclohexyl derivative 21, an analog of Cpd 16 where the basic NH moiety of piperidine ring was replaced with a CH_2_- group was also inactive, reinforcing the importance of a basic center (basicity) for the activity of this class of compounds. 

Next, we initiated target engagement and target deconvolution tasks using label-free techniques and chemical probes, respectively, in order to identify the molecular target(s) responsible for the observed anti-CSC phenotype of Series 1 compounds. Cpd 2 was selected as a starting Cpd for both of these activities primarily due to its easier synthetic accessibility. Before proceeding, however, we first tested the anti-CSC activity of Cpd 2. We observed that Cpd 2, like Cpd 1, reduced the percentage of CD133+ cells and tumor growth in nude mice (Figure S3 and Figure 3E,F), highlighting its anti-CSC properties. Next, we performed a kinome screen to determine if Cpd 2 interacted with kinases, some of which are known to be involved in CSC biology and if targeted can have potential anti-CSC activity. The in vitro binding activity of Cpd 2 against a panel of 468 kinases (KINOMEscan^®^, San Diego, CA, USA) at 1 µM concentration was assessed. The compound turned out to be highly selective, showing a selectivity index S(35) of 0.01. Only 4 kinases were significantly bound: HASPIN (30% signal versus control), the non-receptor tyrosine-protein kinase 2 (TYK2) (12%), serine/threonine-protein kinase pim-3 (PIM-3) (18%) and PIM-1 (5%). After dose response characterization using kinase orthogonal assays, the compound showed significant activity for PIM-1,-3 and HASPIN (IC_50_ = 98, 129 and 915 nM, respectively) and very weak inhibition of TYK2 (IC_50_ = 65 µM) (Figure S4A and Table S1). However, we discarded PIM-1,-3 and HASPIN as the relevant targets of Cpd 2 against CSCs because compound Cpd 1 is not a PIM-1,-3 nor a HASPIN inhibitor (IC_50_ > 10 µM for all; Figure S4B). These results pointed to a non-kinase target as responsible for the anti-CSC effects of Series 1 compounds.

**Figure 4:**
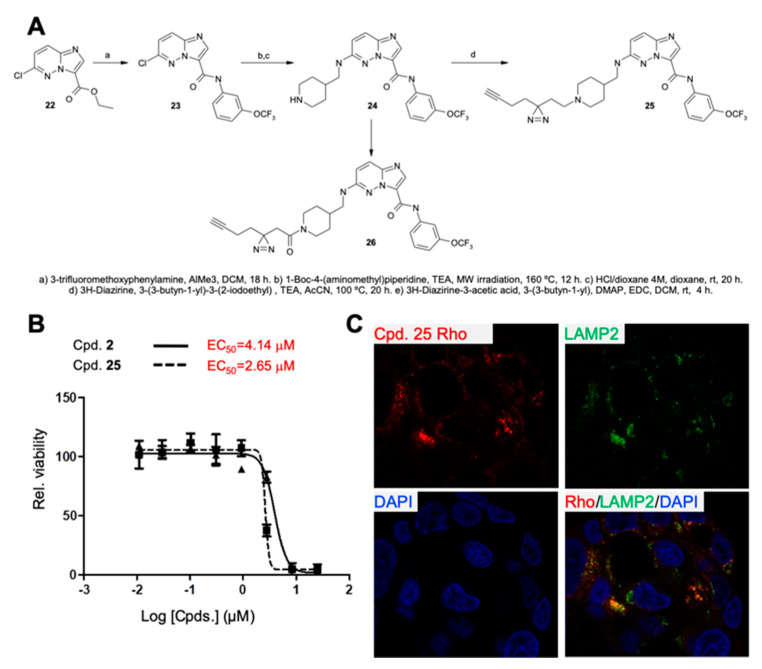

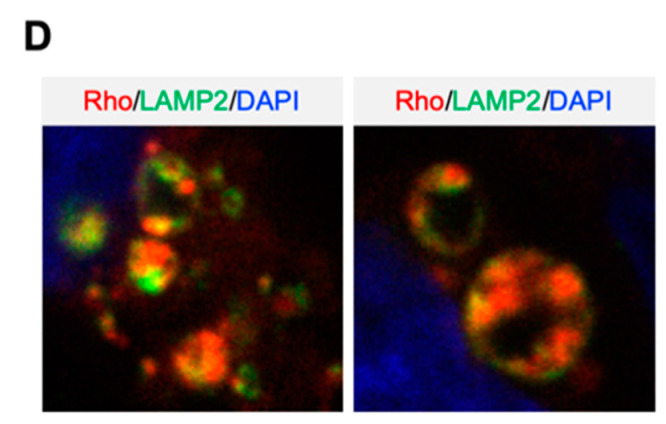
Chemical probes of Series 1 demonstrate interaction with lysosomes. (**A**) Synthesis of PAL probe Cpd. **25**. (**B**) Viability curves performed on PDAC4 3D spheres treated with increasing concentrations of Cpds. 2 or 25 for 30 h. Indicated in red are the calculated EC_50_. (**C**) Fluorescence analysis of Rhodamine-conjugated (via click chemistry) Cpd. 25 localization in PDAC4 cells. Cells were additionally stained with an anti-lysosome-associated membrane protein 2 (LAMP2) antibody and DAPI. LAMP2 localization was visualized with an Alexa-fluor 488-labelled secondary antibody (10×). (**D**) Representative amplified images of Rhodamine localization with LAMP2-positive vesicles (Adobe^®^ Photoshop^®^ CS5 extended v11.0, San Jose, CA, USA).

Next, a chemical proteomics approach based on the design and use of Photo-Affinity Labeling (PAL) chemical probes derived from Cpd 2 was attempted. To this end, we designed an irreversible PAL chemical probe—Cpd 25—based on Cpd 2 and its non-basic amide inactive analogue Cpd 26. A so-called “minimalist linker” with a photoactivable diaziridine moiety and an alkyne reporter tag was incorporated into Cpd 2. The diazirine group of 25 would allow, after incubation with cells and short UV irradiation, the generation of highly reactive carbene species, which would react covalently with those proteins bound or very proximal to the probe. On the other hand, the terminal alkyne group of 25 could be used for subsequent conjugation to suitable reporters (rhodamine-N_3_ or biotin-N_3_) using bio-orthogonal click chemistry. These conjugated molecules were then used to determine the localization of the probe in cells by imaging techniques and in pull-down experiments to identify probe-linked cellular protein targets [29]. The PAL probe 25 was synthesized following standard procedures (Figure 4A). Next, the effect of Cpds 2 and 25 (and the inactive Cpd 26) on PDAC4 CSC viability was assessed side by side, and the activity of probe 25 was comparable or even better than that of Cpd 2 (EC_50_ = 2.62 and 7.17 μM, respectively, Figure 4B), whereas, as expected, Cpd 26 was inactive. 

Probe 25 was then used to determine intracellular localization. Specifically, 7-day-old PDAC2 spheres re-plated as adherent for 24 h were treated with Cpd 25, exposed to UV to crosslink the probe to its potential putative targets and/or proximal proteins, and subsequently conjugated to rhodamine via click chemistry to visualize the probe. Confocal microscopy imaging revealed that Cpd 25 concentrated in vesicular structures reminiscent of lysosomes. Co-staining of cells for the lysosome-associated membrane protein 2 (LAMP2), a marker of lysosomes, confirmed that Cpd 25 concentrated close to LAMP2-positive structures, appearing to localize within lysosomes (Figure 4C,D). 

### 2.4. Compound 2 Induces Lysosomal Membrane Permeabilization

**Figure 5:**
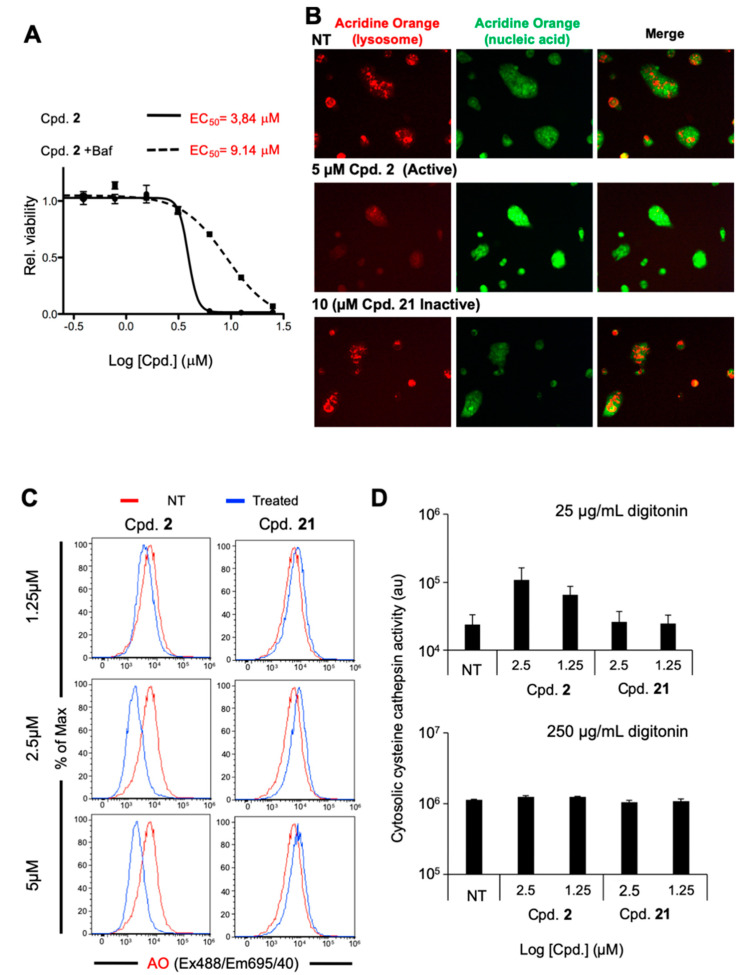
Compound 2 induces lysosomal membrane permeabilization. (**A**) Viability curves performed on PDAC2 3D spheres either untreated or pretreated with 100 nM Bafilomycin for 5 h, then treated with increasing concentrations of compound Cpd. 2 for 72 h. Indicated in red are the calculated EC_50_. (**B**) Fluorescence microscopy analysis of Acridine Orange in re-plated PDAC2 spheres. Cultures were loaded with 1 µg/mL acridine orange for 15 min then non-treated (NT) or treated with either Cpd. 2 (5 µM) or Cpd. 21 (10 µM) for 2 h. Fluorescence was captured in the red and green channels to measure the amount of acridine orange staining acidic organelles or nucleic acids, respectively (Nikon Eclipse T*i*, 10×). (**C**) Representative flow cytometric analysis of Acridine Orange (AO) fluorescence in re-plated PDAC2 spheres. Cultures were either non-treated (NT) or treated with 1.25, 2.5 or 5 µM of Cpd. 2 or Cpd. 21, and fluorescence was assessed at 24 h post treatment. (**D**) Cytosolic cysteine cathepsin activity, expressed as arbitrary units (au), was determined in PDAC2-derived spheres. Spheres were either NT or treated with 1.25 or 2.5 µM of Cpd. 2 or Cpd. 21 for 6 h then permeabilized with 25 µg/mL digitonin (top) to assess cytosolic cathepsin activity or 250 µg/mL digitonin to assess total cellular cathepsin activity (bottom).

Given that the localization of Cpd 2 to lysosomes appeared to be required for its activity, we considered that it might be inducing lysosomal membrane permeabilization (LMP) as a mechanism to reduce CSC viability. It is known that LDCD-mediated cell death can be produced by lysosomotropic drugs. The accumulation of these agents in the lysosome is dependent on their lipophilicity and basicity, both of which are essential for their interaction with the lysosomal membrane, protonation in the acid lysosomal environment, and subsequent retention within lysosomes [30]. As described above, the anti-CSC activity of Cpd 2 also depends on its basicity (pKa = 10.01) and its lipophilicity (cLogP = 4.04), which falls within the accepted range for lysosomotropic agents. The same behavior was observed for other active analogues in this series (Figure S2). Therefore, to test this hypothesis, we first confirmed that the effect of Cpd 2 on PDAC2 CSC survival was partially prevented (≈ 3-fold) by the addition of Bafilomycin-A (Figure 5A), a V-ATPase inhibitor, which increases the pH of the lysosome (less acidic) thereby, presumably reducing the accumulation of Cpd 2 in lysosomes. 

Next, we explored whether Cpd 2 could induce canonical characteristics of LMP by using a standard assay involving loading cells with acridine orange (AO), a fluorescence dye used to stain acidic vacuoles. Re-plated PDAC2-derived sphere cultures were loaded with AO and subsequently treated with either Cpd 2 or negative control Cpd 21, and AO fluorescence was visualized by fluorescence microscopy. As expected, untreated cells showed a vesicular pattern of AO staining in the far-red channel, reflecting accumulation in lysosomes, and a diffuse cytosolic and nuclear staining in the green channel, reflecting AO’s known ability to bind nucleic acids (Figure 5B). Treatment of cells with 5 µM of Cpd 2 greatly reduced lysosomal staining in the far-red channel within just 2 h and significantly increased the overall signal in the green channel, a phenomenon indicative of LMP (Figure 5B). As expected, cells treated with the non-active Cpd 21 had the same pattern of staining in both the red and green channels as untreated cells, even at a dose of 10 µM, indicating that the activity of Cpd 2 against CSC viability and lysosomal integrity were linked. Flow cytometric analysis performed on PDAC2 cells from a parallel experiment quantitatively confirmed these results at 6 h (Figure S5A) and 24 h (Figure 5C) post treatment, with Cpd 2 decreasing overall AO mean far-red fluorescence intensity (MFI) in treated cells and with Cpd 21 having no effect on MFI. Similar affects were observed with PDAC1, PDAC3 and PDAC4 cells with Cpd 2 at 5 µM at 24 h (Figure S5B). We additionally assessed the effect of Cpd 2 on cytosolic cysteine cathepsin activity, another standard assay for monitoring the release of lysosomal contents after LMP. Consistent with the microscopy- and cytometry-based results, treatment of re-plated PDAC2-derived spheres with Cpd 2 increased cytosolic cysteine cathepsin activity, while Cpd 21 had no effect (Figure 5D). 

### 2.5. Broad Spectrum of LMP-Inducing Agents Target CSCs

**Figure 6:**
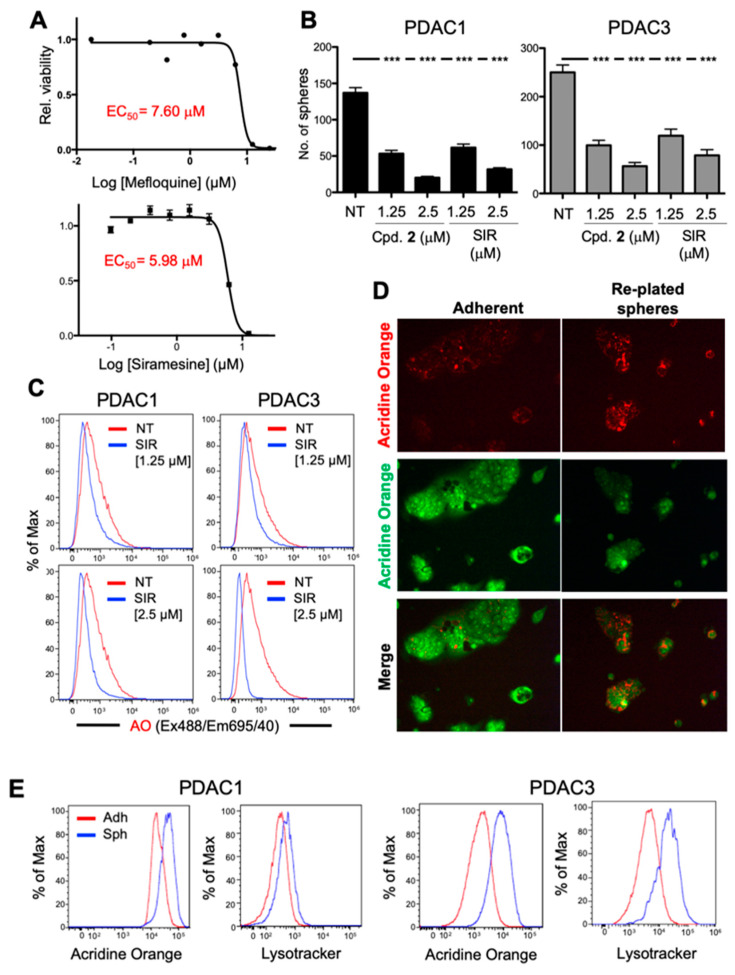
Lysosomal membrane permeabilization (LMP)-inducing agents specifically target CSCs. (**A**) Viability curves performed on PDAC2 3D spheres treated with increasing concentrations of mefloquine (top) or siramesine (bottom) for 30 h. Indicated in red are the calculated EC_50_. (**B**) Number of 1st generation spheres/mL ± standard deviation for PDAC1 (left) or PDAC3 (right) cultures treated with Cpd. 2 or siramesine (SIR), both at 1.25 or 2.5 µM (*n* = 6; Student’s t-test, *** *p* < 0.001). (**C**) Representative flow cytometric analysis of Acridine Orange (AO) fluorescence in re-plated PDAC1 or PDAC3 spheres. Cultures were either non-treated (NT) or treated with 2.5 µM of SIR, and fluorescence was assessed at 24 h post treatment. (**D**) Fluorescence microscopy analysis of Acridine Orange in adherent and re-plated PDAC2 spheres. Cultures were either non-treated (NT) or loaded with Acridine Orange for 15 min. Fluorescence was captured in the red and green channels to measure Acridine Orange in acidic compartments or binding nucleic acids, respectively (Nikon Eclipse T*i*, 10×, Amsterdam, Netherlands). (**E**) Representative flow cytometric analysis of Acridine Orange or Lysotracker fluorescence in PDAC1 or PDAC3 adherent or sphere cultures treated with each probe for 24 h.

In accordance with evidence indicating that glioblastoma and leukemic CSCs present an enhanced susceptibility to LMP-inducing agents [19,20], well-established LMP-inducers mefloquine and siramesine also affected PDAC2 CSC viability with varying potencies (Figure 6A). We next assessed the effect of siramesine (SIR) alongside Cpd 2 on the self-renewal/sphere-forming capacity of PDAC1 and PDAC3 cultures, and observed that both compounds decreased sphere forming capacity at both concentrations tested (Figure 6B). Like Cpd 2, we confirmed that the anti-CSC effect of siramesine is associated with LMP activity (Figure 6C). 

Based on the sum of these results, we speculated whether pancreatic CSCs might have an increased lysosomal mass compared to non-CSCs, which might account for their increased sensitivity to LMP-inducing agents. Indeed, altered lysosomal biology has been reported in CSCs from other tumor types such as breast and embryonal rhabdomyosarcoma [31,32]. To explore this possibility, we loaded re-plated PDAC2-derived spheres and PDAC2 adherent cell populations with AO and observed a clear increase in lysosomal mass in re-plated PDAC2-derived spheres compared to adherent cells, as evidence by an increased AO signal in the far-red channel relative to the green (Figure 6D). This observation was validated by flow cytometry (Figure 6E), using AO and lysotracker. Moreover, we took advantage of our previously published RNA sequencing data (ArrayExpress: E-MTAB-3808 [33]) to confirm whether CSC-enriched sphere cultures over express a previously reported lysosomal biogenesis gene signature [34] compared to their adherent counterpart cultures. As expected, CSC-enriched sphere cultures showed transcriptional upregulation (Figure S6A) and significant enrichment (Figure S6B) of this gene signature set. Together, these data suggest that Cpd 2 is a novel LMP-inducing agent.

### 2.6. LMP Inhibits Pancreatic CSC-Mediated Tumorigenesis and Disease Recurrence In Vivo

**Figure 7:**
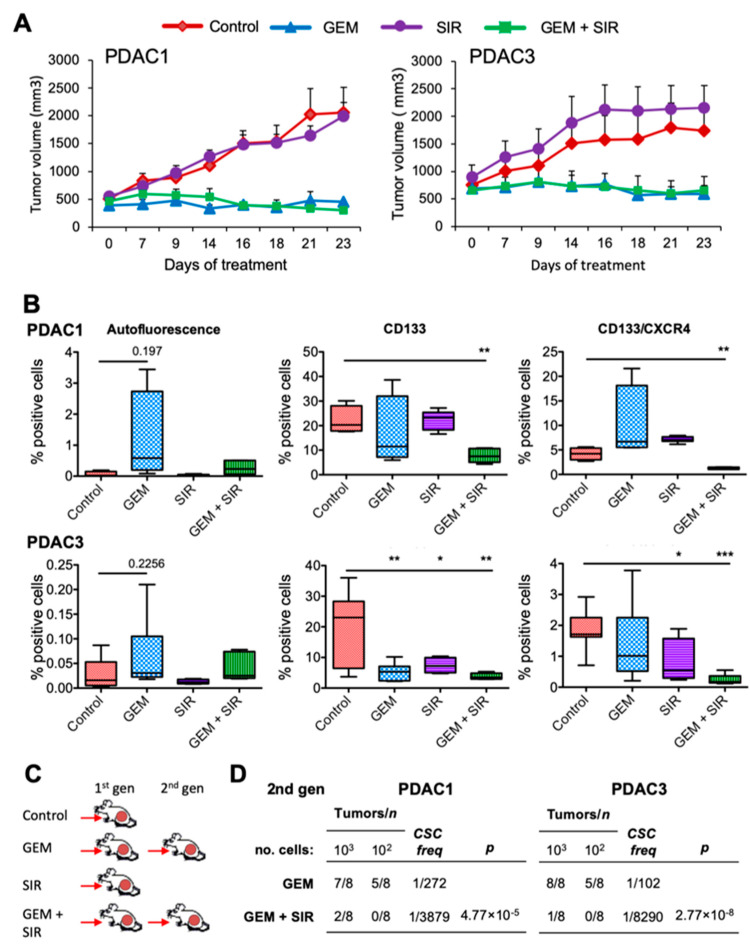
LMP inhibits pancreatic CSC-mediated tumorigenesis and disease recurrence in vivo. (**A**) Change in tumor volume (mm^3^) in PDAC1 and PDAC3 xenografts over the course of 23 days. Mice were treated with diluent (Control), gemcitabine (GEM; biweekly 125 mg/kg i.p.), siramesine (SIR; 30 mg/kg daily oral gavage) or GEM+SIR. (**B**) Summary of flow cytometric analysis of CSC markers: autofluorescence, CD133 or CD133/CXCR4 in PDAC1 and PDAC3 tumors resected on day 23 post treatment initiation (Student’s t-test). * *p* < 0.05; ** *p* < 0.01; *** *p* < 0.001. (**C**) Diagram of serial passage strategy to establish 2nd generation (gen) tumors in vivo. (**D**) Summary of number of tumors obtained/number of injections from GEM and GEM+SIR serial passaged 1st gen tumors. CSCs frequencies and *p* values were calculated using the Extreme Limiting Dilution Analysis software.

Lastly, we wanted to translate these findings to human patient-derived xenografts (PDX) in vivo. Initial in vivo PK studies of Cpd 2 in BALB/c mice showed high clearance (>100% hepatic blood flow) and low levels in plasma after intravenous administration pointing to high rate of drug elimination (Figure S7A,B). Given the poor PK properties of Cpd 2, the well-known orally bioavailable LMP-inducer siramesine was selected as a tool compound to test the proof of concept that LMP-inducing agents can impact PDAC CSC biology in vivo. It is important to note the good pharmacokinetics of siramesine, non-toxic effects in humans and the well-established mechanism of action as an LMP [35,36]. Moreover, siramesine and Cpd 2 showed, in our hands, comparable anti-CSC activity in vitro (Figure 6B). To this end, we implanted nude mice with PDAC1 or PDAC3 PDXs. Once tumors reached 500 mm^3^, mice were randomized to one of the following four treatment groups: Diluent control; Gemcitabine (GEM) (biweekly 125 mg/kg i.p.); siramesine (SIR) (30 mg/kg oral gavage); or GEM + SIR (Figure S7C). Interestingly, for both utilized PDX models, no significant differences were observed for vehicle-treated and SIR single treatment; however, tumors treated with GEM or a combination of GEM + SIR were significantly reduced compared to control tumors and single treatment tumors (Figure 7A). Importantly, flow cytometry analysis of dissociated tumors at day 23 post treatment showed differences in the percentage of cells expressing the CSC markers autofluorescence, CD133 or CD133/CXCR4 following treatment. While not significant, for PDAC1 there was a marked increase in autofluorescent-positive cells with GEM treatment, indicating an enrichment in this population

On the contrary, both the CD133+ or CD133+/CXCR4+ CSC populations significantly decreased only in the combination therapy group (Figure 7B). A similar trend was observed with PDAC3 (Figure 7B). Since our analyses of the percentage of CSCs in tumors following treatment revealed that only combination therapy significantly reduced the CSC population, we injected equal numbers of cells from dissociated GEM-treated and GEM+SIR-treated tumors into recipient mice to establish secondary tumors as a means of measuring tumor relapse potential (Figure 7C). Eight weeks post injection, cells derived from tumors treated with GEM showed enhanced tumor take and increased CSC frequency (1 in 272 for PDAC1 and 1 in 102 for PDAC3) compared to cells derived from tumors treated with GEM+SIR (1 in 3879 for PDAC1 and 1 in 8290 for PDAC3) (Figure 7D). Taken together, these data demonstrate that while GEM alone can reduce tumor size, the addition of a CSC-specific therapy (i.e., SIR) is necessary to eliminate the CSC population and subsequent tumor relapse potential. 

## 3. Discussion

From a clinical perspective, eradication of the CSC compartment should lead to tumor elimination; however, targeting CSCs has proven difficult and technically challenging, and to date few anti-CSC therapies exists. Unfortunately, and as discussed above, screening platforms to discover new anti-CSC therapies have been hampered by the lack of in vitro systems that are sufficiently enriched in CSCs to facilitate the identification of “true” anti-CSC compounds. Consequently, the use of surrogate models of CSCs are becoming increasingly more common. For example, human pluripotent stem cell- and mESC-based platforms have been shown to faithfully reproduce properties of somatic CSCs [37,38,39,40]. Based on these observations and the fact that in general mESCs share many properties with CSCs, including self-renewal and expression of pluripotency-associated genes [8], we used mESCs as a surrogate system for CSCs in our small-scale screen to identify a unique class of small-molecules with potent anti-pancreatic CSC properties.

Using this approach, two classes of compounds were identified in the initial screen using mESCs. Series 2 contained PI3K and/or mTOR kinase activity inhibitors, and while this series was not further explored in this study, their identification confirmed the effectiveness of the screen as PI3K/mTOR signaling is important for CSC biology [21,22], and inhibitors of this pathway are currently being developed to target CSCs [23,24,25,26]. Series 1 contained 5 structurally similar imidazo[1,2-b]pyridazine-3-carboxylic acid (3-trifluoromethoxy-phenyl) amide derivatives with a basic nitrogen residue at C-6 position of the imidazo[1,2-b]pyridazine or imidazo[1,2-a]pyrazine scaffolds. Importantly, not only were Series 1 compounds effective against mESCs, but they faithfully recapitulated their activity in primary human PDAC cultures, derived from 4 different PDX models, showing potent and specific anti-CSC activity. Specifically, we demonstrated that the identified compounds preferentially targeted pancreatic CSCs over non-CSCs as Series 1 compounds were more effective in cultures enriched in CSCs (e.g., 3D spheres or 2D re-plated spheres) and cells expressing known pancreatic CSC markers, such as CD133 or autofluorescence, decreased in percentage and preferentially entered into apoptosis upon treatment with low micromolar doses. The loss of the CSC compartment in vitro translated into an ablation or significant reduction in the in vivo tumorigenic potential of cells pre-treated with these compounds, specifically with Cpd 1 or Cpd 2, respectively. The complete opposite was seen with Gemcitabine, which we have previously shown enriches for CSCs [28,41]. 

Having discarded their interaction with kinases via a KINOMEscan, Photo-Affinity Labeling (PAL) chemical probes, with a photoactivable diaziridine moiety and an alkyne reporter tag, were utilized to identify proteins bound or proximal to the probe or to visualize the probe via fluorescence microscopy, respectively, as a means of elucidating the anti-CSC mechanism of action of these compounds. This approximation showed that the Series 1 compounds had a clear affinity for the lysosome. In addition, we determined that the presence of the basic nitrogen residue at C-6 position was necessary for the activity of Series 1 compounds, as non-basic derivatives (pKa < 7) showed no anti-CSC activity. For example, the anti-CSC activity of Cpd 2 depended on its basicity (pKa = 10.01) and its lipophilicity (cLogP = 4.04), both of which fall within the accepted range for LMP inducers. One of the best studied LMP inducers is the σ 2 receptor agonist, siramesine (Lu-28–179; 1’-[4-[1-(4-Fluorophenyl)-1H-indol-3-yl]butyl]spiro[isobenzofuran-1(3H),4’-piperidine). While initially designed to treat anxiety and depression [42], it was shown to be a more potent LDCD inducer, destabilizing the lysosomal membrane to promote the release of cathepsins resulting in cathepsin-mediated death in tumor cells [43,44]. In 2017, Jensen et al., tested the anti-CSC effects of siramesine on glioblastoma in vitro and in vivo and showed that siramesine had potent effects on glioma cell lines and patient-derived spheroids cultures enriched in CSCs in vitro [20]. Another recently described LMP inducer is mefloquine, a quinoline used for the treatment and prevention of malaria [45], but which was shown to induce LDCD in human acute myeloid leukemia (AML) cells including AML CSCs in vitro and in vivo [19]. It is important to note that the aforementioned studies represent the only two studies to date demonstrating that siramesine or mefloquine can target CSCs by inducing LDCD. While other studies have shown that siramesine can impact breast, lung, cervix, prostate and connective tissue tumor cell lines, the effect on CSCs was not explored in these studies [43,44,46]. 

The accumulation of LMPs in the lysosome is dependent on their lipophilicity and basicity (cLogP > 2 and 6.5 < pKa < 11) [30]. Specifically, the lipophilicity of these agents is essential to facilitate their interaction with the membranes of and entry into lysosomes and their basic center(s) are crucial for their retention in the lysosome following their protonation by the acidic environment of the organelle (pH: 4.5–5.0) leading to their subsequent accumulation. Based on the similarities in basicity and lipophilicity with siramesine and mefloquine and the localization of our compounds with the lysosome in PDAC CSCs, we hypothesized and experimentally confirmed via numerous approaches that our Series 1 compounds were also acting via an LCDC-based mechanism of action. Moreover, when compared to the known LMP inducer siramesine, Cpd 2 showed equivalent anti-CSC activity, confirming that the Series 1 compounds function by inducing LCDC. The aforementioned points have several implications. Firstly, using an independent and unrelated screening approach, we also identified LMP inducers as potent inhibitors of CSCs. As mentioned above, only two other studies to date have identified that LMP inducers can target CSCs of glioblastoma [20] and AML [19]. Importantly, our study expands this observation to PDAC CSCs and highlights that LMP inducers may represent a potential anti-CSC therapy for many tumor types (both solid and non-solid). Secondly, siramesine showed equivalent anti-CSC activity as Cpd 2 in vitro. Furthermore, when siramesine was used together with gemcitabine in two PDX models, combination treatment efficiently and significantly reduced the CSC compartment, which was validated by flow cytometry and in serial tumorigenesis assays where the CSC frequency was reduced to 1 in 8290. While gemcitabine alone equally reduced PDAC1 and PDAC3 tumor growth in first generation tumors, flow cytometry analysis of these tumors confirmed an enrichment rather than a reduction in the CSC compartment, which resulted in tumor relapse upon serial passage. Siramesine alone had no effect on tumor size, but did reduce the percentage of CD133^+^ and CD133^+^/CXCR4^+^ in PDAC3 tumors; however, this effect was not sufficient to reduce tumor growth at the dosage used (30 mg/kg). Thus, in the case of LMP inducers, higher doses or targeting both the CSC and the more differentiated bulk tumor cells (e.g., with gemcitabine) would likely be necessary to achieve sustained tumor clearance. It is interesting that Jensen et al. similarly showed that siramesine alone was ineffective in vivo (with 100 mg/kg) using implanted invasive glioblastoma spheroids or gliobastomas PDXs [20]; however, the authors did not use siramesine in combination with standard chemotherapeutics, nor did they examine the CSC compartment or perform serial transplantation analysis. Thus, the ability of siramesine to effectively target glioblastoma CSCs is still unresolved and would benefit from combination therapy-based studies. Lastly, while we did not measure lysosomal size, we did show that PDAC CSCs have increase lysosomal mass. The latter we attribute to increased lysosomal biogenesis, which was confirmed by RNAseq analysis using a previously published lysosomal biogenesis gene signature set [33]. Using this same signature, Sukhai et al. demonstrated a similar phenotype for primitive and progenitor AML CSCs, which they successfully targeted with mefloquine [19]. Taken together, these observations indicate a plausible mechanism to explain the observed differential and specific activity of the LMP inducers siramesine or mefloquine at the level of CSCs, at least for AML and PDAC, respectively. It would not be surprising if increased lysosomal mass is a conserved hallmark of CSCs across different tumor entities, which is necessary for their biological activity and “stem”-ness. Thus, targeting the lysosome may represent a true Achilles’ heel for CSCs in general and should be further explored. 

## 4. Materials and Methods 

### 4.1. CNIO-640 Chemical Library

The Experimental Therapeutics Program at the CNIO, ETP-CNIO, owns a chemical library of about 50,000 single compounds built as a result of the consolidation of several sub-libraries selected attending to different criteria such as chemical diversity, kinase-targeted focus, potential to disrupt protein–protein interactions, and the presence of low molecular weight compounds to facilitate fragment-based drug discovery. The drug-likeness of the whole library was also ensured by the application of filters such as “rule of five” [47]. The compounds were selected from commercial origin as well as from internally newly designed and synthesized chemical matter. Representative libraries of the whole 50,000 library with smaller sizes were defined after clustering, based on similarity analysis, and selection of representative compounds from each cluster. A 640-compound library, subject of the current detailed screening campaign, is the minimum size set of compounds representing the chemical ETP-CNIO collection. 

### 4.2. Murine Embryonic Stem Cells

Mouse embryonic stem cells were cultured in Dulbecco’s Modified Eagle Medium (DMEM) containing 15% Fetal bovine serum (FBS), non-essential amino acids (NEAA), 2-mercaptoethanol, and Leukemia inhibitory factor (LIF) and passaged on gelatinized plates under standard conditions.

### 4.3. Primary Human Pancreatic Cancer Cells

The PDAC patient-derived xenograft (PDX) Panc354 (referred to hereafter as PDAC3) was obtained from Dr. Manuel Hidalgo under a Material Transfer Agreement with the Spanish National Cancer Centre (CNIO), Madrid, Spain (Reference no. I409181220BSMH). PDAC1, PDAC2 and PDAC4 PDXs were established from primary patient resected tumors. Samples were provided by the BioBank Hospital Ramón y Cajal-IRYCIS (PT13/0010/0002), integrated in the Spanish National Biobanks Network and they were processed following standard operating procedures with the appropriate approval of the Ethical and Scientific Committees (Control no. No. Control: DE-BIOB-73 AC65, RG.BIOB-96 REV, and CI-BIOB-08-10-01 rev), with informed consent and according to Declaration of Helsinki principles. Primary patient tumors were subcutaneously implanted in immunocompromised female 6- to 8-week-old NU-Foxn1nu nude mice (Envigo, Barcelona, Spain) and subsequently passage in vivo to establish PDX PDAC1, PDAC2 and PDAC4. Primary PDX-derived in vitro cultures were established as previously detailed [48]. Briefly, xenografts were minced, enzymatically digested with collagenase (Stem Cell Technologies) for 60 min at 37 °C and after centrifugation for 5 min at 1800 rpm, the cell pellets were resuspended and cultured in RPMI (1:1; #61870-010; Gibco, Waltham, MA, USA) supplemented with 10% FBS (Invitrogen, Waltham, MA, USA), Pen/Strep (1:100; #1500-063; Gibco, Waltham, MA, USA), and Fungizone (1:250; #15290-018; Gibco, Waltham, MA, USA). Primary cultures were tested for Mycoplasma at least every 4 weeks. 

### 4.4. Sphere Formation Assay 

Pancreatic CSC spheres were generated as previously described [48] in ultra-low attachment plates (Corning) using CSC media: serum-free DMEM/F12 medium (1:1; #21331-020; Gibco, Waltham, MA, USA), supplemented with Pen/Strep (1:100; #1500-063; Gibco, Waltham, MA, USA), Fungizone (1:250; #15290-018; Gibco), L-Glutamine (1:100; #25030-024; Gibco, Waltham, MA, USA), B-27 (1:50; #17504-044; Gibco, Waltham, MA, USA) and basic fibroblast growth factor (FGF-b) (1:5000; Gibco, Waltham, MA, USA). All cell culture was carried out at 37 °C in a 5% CO_2_ humidified incubator. To quantify spheres of > 40 μm, 1 mL of sample volume was analyzed with a CASY® Cell Counter (Roche Applied Sciences, Mannheim, Germany). The CASY® Cell Counter measuring principle is based on a capillary particle counter with pulse area analysis, that permits the determination of cell count, cell concentration, cell volume (peak), and average cell diameter, specifically diameters of 40–80 μm, 80–120 μm and >120 μm.

### 4.5. Drug Screening and Cell Viability Assays

Murine embryonic stem cells (mESCs) were plated at 20,000 cells per well in gelatinized 96 well plates. Compounds from the 640 compound ETP library were added by a Beckman FX 96 tip to achieve a final concentration of 5 µM in each well, and each compound was tested in duplicate. Cells were treated for a total of 30 h prior to assessing cell viability using CellTiter 96^®^ AQueous One Solution Cell Proliferation Assay (MTS) assay according to manufacturer’s instructions (G3582, Promega, Madison, WI, USA). Dose response curves were carried out on mESCs under the same conditions using indicated compounds. To test the viability of CSCs, 10,000 PDAC cells were plated in spheroid conditions and immediately treated with indicated compounds and concentrations for the duration of the sphere formation assay. For adherent non-CSCs, 20,000 cells were plated in wells of standard tissue culture-treated plates (Corning, Corning, NY, USA) and treated the following day with indicated compounds and concentrations. For re-plated spheres (Figure S1B), 7-day-old spheres were filtered using a 40 µM filter (Fisher Scientific, Hampton, NH, USA), washed once with 1× PBS, and incubated with trypsin 0.05% EDTA at 37 °C (Thermo Fisher Scientific, Waltham, MA, USA) to achieve a single cell suspension (approximately 10–30 min, depending on the PDAC culture). Trypsinized cells were counted and 20,000 cells were plated in wells of standard tissue culture-treated plates (Corning) and treated the following day with indicated compounds and concentrations.

### 4.6. Flow Cytometry

Cells were analyzed with a 4-laser Attune NxT Acoustic Cytometer (ThermoFisher Scientific). Cells and digested tumors (as described in [41]) were resuspended in FLOW buffer (1X PBS; 3 mM EDTA (v/v); 3% FBS (v/v)), and the following fluorescently-tagged antibodies were used to label cells for 30 min at 4 °C: Mouse monoclonal anti-human CD133-PE (1:20, Cat no. 130-110-962, Miltenyi, Bergisch Gladbach, Germany), Mouse monoclonal anti-human CD133-APC (1:20, Cat no. 130-111-080, Miltenyi, Bergisch Gladbach, Germany), mouse monoclonal anti-human C-X-C chemokine receptor type 4- Phycoerythrin (CXCR4-PE) (1:20, Cat no. 130-117-354, Miltenyi, Bergisch Gladbach, Germany), or mouse monoclonal anti-human Epithelial cell adhesion molecule-fluorescein isothiocyanate (EpCAM-FITC) (1:20, Cat no. 130-111-115, Miltenyi, Bergisch Gladbach, Germany). Autofluorescent cells were excited with blue laser 488nm and selected as the intersection with the filters 530/40 and 580/30 as previously described [27]. For lysosomes quantification, Acridine Orange (AO) (A1301, Thermo Fisher Scientific, Waltham, MA, USA) or Lysotracker Deep Red (DR) (L12492, Molecular probes, Life Technologies, Waltham, MA, USA) were used. AO fluorescence was assessed with two combinations of excitation and emission spectra: BL1 (Ex488/Em695/40, equal to the far-red channel (>670 nm)) and BL3 (Ex488/Em695/40, equal to the far-red channel (>670 nm)) Lysotracker DR fluorescence was assessed with RL1 (Ex638/Em670/14). Bafilomycin A1 (100 nM, Calbiochem, San Diego, CA, USA) was included as a positive control for lysosome disruption. For Annexin V staining, floating and attached cells were pooled and resuspended in 1× AnnexinV staining buffer containing AnnexinV-APC diluted 1:20 (Biotium, Freemont, CA, USA) and incubated for 20 min at room temperature prior to flow cytometric analysis. For all assays, DAPI was used to mark and exclude dead cells, and data were analyzed using the software FlowJo v9.3 (Tree Star Inc., Ashland, OR, USA).

### 4.7. In Vivo Assays

Female 6- to 8-week-old NU-Foxn1nu nude mice (Envigo, Spain) were subcutaneously injected with control or pre-treated 1 × 10^4^ PDAC cells in 50 µL of Matrigel^TM^ (Corning). Tumor growth was monitored bi-weekly for up to 10 weeks. For Siramesine experiments, mice were subcutaneously implanted with PDAC1 and PDAC3 PDXs. After 4 weeks of tumor growth, mice were randomized into treatment groups (5 mice per group). Mice were treated with Siramesine twice a week (30 mg/kg by oral gavage formulated in 0.5% methylcellulose 15 cP in 0.9% NaCl solution) for ~4 weeks. Gemcitabine (GEMZAR^®^, Eli Lilly, Indianapolis, IN, USA) was administered twice a week (125 mg/kg i.p.) for ~4 weeks. Tumor volumes (mm^3^) were determined twice per week using a digital caliper. Mice were sacrificed at 24 days post treatment initiation and tumors were extracted and analyzed by flow cytometry as described above. For second generation tumor formation, 1000 and 100 cells of indicated treatment groups were resuspended in Matrigel ^TM^ and injected subcutaneously in recipient NU-Foxn1nu nude mice and tumor formation was monitored for 10 weeks. Mice were housed according to institutional guidelines and all experiments were performed in compliance with the institutional guidelines for the welfare of experimental animals approved by the Universidad Autónoma de Madrid Ethics Committee (CEI 60-1057-A068) or by the Instituto de Salud Carlos III Ethics Committee (CBA12_2014-v3) and La Comunidad de Madrid (PROEX 335/14 or PROEX 53/14) and in accordance with the guidelines for Ethical Conduct in the Care and Use of Animals as stated in The International Guiding Principles for Biomedical Research involving Animals, developed by the Council for International Organizations of Medical Sciences (CIOMS).

### 4.8. Pharmacokinetic Studies (PK)

Female BABL/c mice, 10 weeks old were used (*n* = 3 per time point). Compound 2 was formulated in 0.9% NaCl solution for both oral and intravenous (i.v.) injections. Plasma samples were collected following a single i.v. or oral administration of 8 mg/kg at 0.08, 0.16, 0.25, 0.5, 1, 4, 8 and 24 h. The extraction of Cpd 2 from plasma was achieved by solid phase extraction followed by high performance liquid chromatography/tandem mass spectrometry (Agilent 1100, Applied Biosystems API2000) analysis. The amount of inhibitor and the internal standard in each mouse plasma sample were quantified based on calibration curves generated using standards of known concentrations of compound. This assay method was sufficiently accurate for the quantification of Cpd 2 with a limit of detection (LOD) and a low limit of quantification (LLOQ) of 5 ng/mL. Pharmacokinetic parameters were estimated using WinNonlin Version 5.2 software (Pharsight Corp., Mountain View, CA, USA), by fitting both the experimental i.v and oral data to a bicompartimental model.

Mice were housed according to institutional guidelines and all experiments were performed in compliance with the institutional guidelines for the welfare of experimental animals approved by the Instituto de Salud Carlos III Ethics Committee (CBA12_2014-v3) and La Comunidad de Madrid (PROEX 53/14), and in accordance with the guidelines for Ethical Conduct in the Care and Use of Animals as stated in The International Guiding Principles for Biomedical Research involving Animals, developed by the Council for International Organizations of Medical Sciences (CIOMS).

### 4.9. KinomeScan

The KINOMEscan ^TM^ screening platform employs an active site-directed competition binding assay to quantitatively measure interactions between test compound and 468 human kinases and disease relevant mutant variants. These assays do not require ATP and thereby report thermodynamic interaction affinities. The assay was performed at DiscoveRx Corporation.

### 4.10. Synthesis of Affinity Chemical Probes

General Procedure; Chemicals were purchased from Aldrich Chemical Company Ltd., Apollo Scientific Ltd. and TCI Europe N.V. Unless otherwise stated, commercial chemicals, and solvents were used without further purification. Anhydrous dichloromethane (DCM), dioxane, acetonitrile, and TEA (Tris base, acetic acid and EDTA) were purchased from Aldrich in Sure SealTM bottle and kept under nitrogen. Proton (^1^H) NMR spectra were recorded on Bruker Avance II 300 using DMSO-d6 as solvent. Chemical shifts are expressed in parts per million (ppm) (δ relative to residual solvent peak for 1 H). Multiplicities are indicated by s (singlet), d (doublet), t (triplet), q (quartet), m (multiplet), br (broad) or combination thereof. 

The high-performance liquid chromatography (HPLC) measurements were performed using a HP 1100 from Agilent Technologies comprising a pump (binary) with degasser, an autosampler, a column oven, a diode-array detector (DAD) and a column Gemini-NX C18 (100 × 2.0 mm; 5 µm particle size). Eluent A, water with 0.1% formic acid; eluent B: acetonitrile with 0.1% formic acid. Gradient 5% to 100% of B within 8 min at 50 °C, DAD. Flow from the column was split to a MS spectrometer. The MS detector was configured with an electrospray ionization source or API/APCI. Nitrogen was used as the nebulizer gas. Data acquisition was performed with ChemStation LC/MSD quad, software. Purities of all reported compounds were greater than 95% based on HPLC chromatograms obtained on an Agilent HP 1100 LCMS system.

6-Chloro-imidazo[1,2-b]pyridazine-3-carboxylic acid (3-trifluoromethoxy-phenyl)-amide (23).

Trimethylaluminium (2M in hexanes, 1 mL, 2 mmol) was carefully added to a solution of 3-trifluoromethoxyphenylamine (345 mg, 2 mmol) in dry CH_2_Cl_2_ (9 mL), under N_2_, and this mixture was stirred for 30 min. Then, ethyl 6-chloroimidazo[1,2-b]pyridazine-3-carboxylate 22 (400 mg, 1.773 mmol) was added and the reaction was refluxed (40 °C) for 17 h. The reaction was left to cool down, quenched with HCl 0.2 M (10 mL) and extracted with CH_2_Cl_2_, and washed with brine. The organic layers were dried (MgSO_4_), filtered and concentrated, affording the desired product 23 as a pale brown solid (530 mg, Y: 83%). LC-MS (ESI) RT 4.3 min m/z 357.1, 359.1 [M+ H]+. 

6-[(Piperidin-4-ylmethyl)-amino]-imidazo[1,2-b]pyridazine-3-carboxylic acid (3-trifluoromethoxy-phenyl)-amide (24).

A solution of compound 23 (194 mg, 0.54 mmol) and 1-Boc-4-(aminomethyl)piperidine (350 mg, 1.632 mmol) in dioxane (7 mL) with triethylamine (0.4 mL) was irradiated in the microwave at 160 °C for 12 h. Water was added and the reaction mixture was acidified to pH 5, then extracted with EtOAc; the organic phase was dried and concentrated to give a residue which was purified by automated chromatography in SiO_2_ (eluents: EtOAc/MeOH 96:4 to 84:16) to give the corresponding tert-butoxycarbonyl (BOC) protected derivative (186 mg, Y: 64%) as a solid. LC-MS (ESI) rt 4.752 min m/z 535.25 [M+ H]+.

The solid in 3 mL of dioxane was treated with 4M HCl/diox (1 mL) and the mixture was stirred at room temperature for 20 h. Ether was added and white solid precipitates were filtered, washed with ether and dried to give compound 24 (194 mg) as a chloridrate salt. LC-MS (ESI) RT 3.093 min m/z 435.15 [M+ H]+.

6-({1-[2-(3-But-3-ynyl-3H-diazirin-3-yl)-ethyl]-piperidin-4-ylmethyl}-amino)-imidazo [1,2-b]pyridazine-3-carboxylic acid (3-trifluoromethoxy-phenyl)-amide (25).

3H-Diazirine, 3-(3-butyn-1-yl)-3-(2-iodoethyl) (45 mg, 0.096 mmol) was added to a solution of compound 24 (47 mg, 0.19 mmol) and TEA (0.08 mL) in AcCN (1 mL) and the mixture was stirred in a closed vessel at 100 °C for 24 h. Aqueous saturated NaHCO_3_ was added and the reaction was extracted with a mixture of CHCl_3_/iPrOH 1:1. The organic phase was dried and concentrated to give a residue which was purified by automated chromatography in SiO_2_ (eluents: DCM/MeOH(NH_3_) 9:1) giving 10 mg of 25 (19% yield). LC-MS (ESI) rt 3.559 min m/z 527.3 mainly fragmentation of diazirine ring [M+ H]+.

^1^H NMR (300 MHz, DMSO): δ ppm 11.07 (s, 1 H), 8-05 (s, 1 H), 7.91 (d, J = 9.6 Hz, 1 H), 7.82 (m, 1H), 7.67 (m, 1 H), 7.61 (m, 1 H), 7.52 (t, J = 8.1 Hz, 1 H), 7.13 (m, 1 H), 6.93 (d, H = 9.9 Hz, 1 H), 3.29 (m, 4 H, signal under water of DMSO), 2. 79 (m, 3 H), 2.07 (m, 2 H), 1.98 (m, 2 H), 1.78 (m, 4 H), 1.54 (m, 4 H).

6-({1-[2-(3-But-3-ynyl-3H-diazirin-3-yl)-acetyl]-piperidin-4-ylmethyl}-amino)-imidazo[1,2-b]pyridazine-3-carboxylic acid (3-trifluoromethoxy-phenyl)-amide (26).

DMAP (20 mg, 0.16 mmol) and EDC (10 mg, 0.05 mmol)) were added to a solution of compound 24 (15 mg, 0.03 mmol) and 3H-Diazirine-3-acetic acid, 3-(3-butyn-1-yl) (10 mg, 0.06 mmol) in dry DCM (1 mL) and the mixture was stirred at room temperature (RT) for 4 h. Solvent was removed and the residue purified by automated chromatography in SiO_2_ (DCM/MeOH up to 9:1) giving a residue which was purified by HPLC to yield 4 mg of desired compound 26 (22% Yield). LC-MS (ESI) RT 5.65 min m/z 569.3 [M+ H]+.

^1^H NMR (300 MHz, DMSO): δ ppm 11.02 (s, 1 H), 8.06 (s, 1 H), 7.92 (d, J = 9.9 Hz, 1 H), 7.87 (m, 1H), 7.70 (m, 1 H), 7.55 (m, 2 H), 7.13 (m, 1 H), 6.93 (d, H = 9.9 Hz, 1 H), 4.35 (m, 2 H), 3.67 (m, 2 H), 2.96 (m, 2 H), 2.80 (m, 1 H), 1.99 (m, 3 H), 1,8 (m, 3 H), 1.61 (m, 2 H).

### 4.11. Kinase Assays

PIM1/3 kinase activities were measured as previously described in Martínez-Gonzalez et al. [49]. HASPIN activity was measured using the commercial ADP GLO assay (Promega), a homogeneous assay measuring ADP accumulation, as a universal product of kinase activity. The assay was done following general manufacturer recommendations and adapting protein to optimal conditions. Kinase buffer was 15 mM HEPES ((4-(2-hydroxyethyl)-1-piperazineethanesulfonic acid), pH 7.4, 20 mM NaCl, 1 mM egtazic acid (EGTA), 0.02% Tween-20, 10 mM MgCl_2_ and 0.1 mg/mL BGG (bovine γ-globulin). The assay to measure autophosphorylation of HASPIN (ProQinase, Freiburg, Germany) was done at 150 μM ATP. In order to calculate the IC_50_ of described compounds, serial 1:5 dilutions were tested. Luminescence counts were read in a Victor instrument (Perkin Elmer) Values were plotted against inhibitor concentration and fit to a sigmoid dose–response curve with activity base from IDBS software. TYK2 kinase assay was performed at ProQinase.

### 4.12. Immunofluorescence with Click Chemistry Probes

Seven-day-old spheres were disaggregated with trypsin and re-plated in chamber slides. One day after plating, cells were treated with 2.5 µM Cpd 25 for 30 min, then media was removed and replaced with ice cold PBS. Cells were exposed to 366 nM UV for 30 min using a hand-held lamp then washed, fixed, permeabilized and washed once again in PBS containing 1% BSA. Click it chemistry was performed using Click-it reaction cocktail from Invitrogen’s Click-it Cell Reaction Buffer Kit (Cat. no. C10269) containing 5 µM Rhodamine-azide for 2 h. Cell were subsequently stained with LAMP2 mouse monoclonal antibody from Abcam and anti-mouse IgG Alexa Fluor 488 and with DAPI. Preparations were mounted and visualized by confocal microscopy (Leica TCS SP5 Confocal Microscope, Wetzlar, Germany).

### 4.13. Acridine Orange Lysosomal Integrity Assay

Re-plated spheres were loaded with 1 µg/mL acridine orange (A1301, Thermo Fisher Scientific) for 15 min then washed 3 times with PBS. Cells were treated with indicated concentrations of compounds for 2 h. Relative intensities of acridine orange binding to nucleic acids versus that contained in lysosomes was observed with an upright fluorescent microscope or via flow cytometry as detailed above. To visualize lysosomal mass in spheres versus adherent cells, acridine orange was added at 1 µg/mL for 15 min and fluorescent signal was observed in absence of compound treatment via flow cytometry as detailed above or with an upright fluorescent microscope (Nikon Eclipse Ti microscope with NIS Elements BR Imaging Software, Amsterdam, Netherlands). 

### 4.14. Measurement of Cytosolic Cathepsin Activity

Spheres were re-plated, and then treated the following day with the indicated concentration of compounds for 6 h in CSC media. Cells were subsequently placed on ice and extracted with either 25 µg/mL or 250 µg/mL of digitonin in 250 mM sucrose, 20 mM HEPES, 10 mM KCl, 1.5 mM MgCl_2_, 1 mM EDTA, 1 mM EGTA, 1 mM pefablock, pH 7.5. The extracts were taken and spun down briefly to eliminate floating cells. Cathepsin activity was measured in a 96 well format by measuring cleavage of zFR-AMC (Z-Phe-Arg-AMC. Z: N-carbobenzyloxy; 7-Amino-4-methylcoumarin), as previously described [50].

### 4.15. RNA Sequencing

Illumina RNA sequencing of five primary PDX-derived PDAC cultures grown as either adherent (Non-CSCs) or anchorage-independent spheres (CSCs), in duplicate, was previously performed [33], and raw data was deposited in ArrayExpress accession number: E-MTAB-3808. Differential expression of genes across the different conditions was calculated with Cuffdiff. Heatmaps showing gene expression levels (FPKM, Fragments per kilobase of transcript per million mapped fragments) for the different samples were drawn with Morpheus, (https://software.broadinstitute.org/morpheus) for a subset of selected genes involved in Lysosome Biogenesis [34]. This signature set was uploaded to the Gene Set Enrichment Analysis (GSEA) module of the Genepattern suite from the Broad Institute and false discovery rate (FDR) < 25% was considered statistically significant.

### 4.16. Statistical Analyses

Results are presented as means ± standard error of the mean (SEM) unless stated otherwise. An unpaired two-tailed t test was performed to determine significance. *p* values < 0.05 were considered statistically significant. All analyses were performed using GraphPad Prism version 6.0 (San Diego, California USA). CSC frequencies and significance were determined using the on-line extreme-limiting dilution assays (ELDA) program (http://bioinf.wehi.edu.au/software/elda/) [51].

### 4.17. Data Availability

Unique identifiers for publicly available datasets are indicated, a list of figures that have associated raw data can be provided, and there are no restrictions on data availability.

## 5. Conclusions

In conclusion, while CSCs represent the root of most tumors, they are not without their weaknesses. In this study, we screened a chemical library to identify candidate anti-CSC molecules and identified several compounds, belonging to the same series, with potent activity against PaCSCs in several PDX culture models. Interestingly, these compounds acted on PaCSCs via induction of LMP, highlighting a potentially important role for lysosomes in PaCSC biology. The latter is supported by the fact that PaCSCs have increased lysosomal mass, a phenotype that makes them more susceptible to LMP inducers and one that appears to be conserved across CSCs of different tumor entities. While our lead compounds are still far from in vivo application, we show that the well characterize LMP-inducer siramesine can efficiently target and eliminate the PaCSC population in mice implanted with pancreatic PDX tumors, demonstrating the potential clinical application of LMP inducers as a promising anti-CSC therapeutic strategy for PDAC. Thus, inducers of LMP, in general, should be explored as possible therapeutics for CSC-driven tumors.

## Supplementary Materials

The following are available online at https://www.mdpi.com/2072-6694/12/7/1790/s1, Figure S1: Compounds preferentially target CSCs, Figure S2: Cellular activity and calculated physico-chemical properties of compounds from Series 1, Figure S3: Compound 2 preferentially targets CD133+ CSCs, Figure S4: Selectivity profile of compound 2, Figure S5: Compound 2 induces lysosomal membrane permeabilization as early as 6 h, Figure S6: PDAC CSCs upregulate genes involved in lysosome biogenesis, Figure S7: In vivo studies; Table S1: Kinase orthogonal assay results.
