# Supplementary material for: Induction of Lysosome Membrane Permeabilization as a Therapeutic Strategy to Target Pancreatic Cancer Stem Cells
Source: Cancers (Basel). 2020 Jul 4;12(7):1790. doi: 10.3390/cancers12071790 (PMC7407272; doi:10.3390/cancers12071790)
Supplement: Supplementary file 1 [file cancers-12-01790-s001.pdf]

cancers

Supplementary Materials

Induction of Lysosome Membrane Permeabilization as a Therapeutic Strategy to Target Pancreatic Cancer Stem Cells

Timothy P. Cash, Sonia Alcalá, María del Rosario Rico-Ferreira, Elena Hernández-Encinas, Jennifer García, María Isabel Albarrán, Sandra Valle, Javier Muñoz, Sonia Martínez-González, Carmen Blanco-Aparicio, Joaquín Pastor, Manuel Serrano and Bruno Sainz Jr.

A

Series 1, Chemically Similar (yellow)

Series 2, PI3K/mTOR inhibitor (grey)

Compound	PI3K_IC$_{50}$ (M)	MTOR_IC$_{50}$ (M)
1	>5.00E-05	>1.00E-05
2	>5.00E-05	>1.00E-05
3	>5.00E-05	>1.00E-05
4	5.84E-11	4.39E-10
5	>5E-05	>1.00E-05
6	2.79E-09	7.69E-08
7	1.54E-08	1.23E-07
8	2.56E-09	4.43E-07
9	9.04E-11	1.73E-09
10	1.00E-08	4.31E-07
11	>5.00E-05	>1.00E-05
12	8.25E-09	6.66E-08

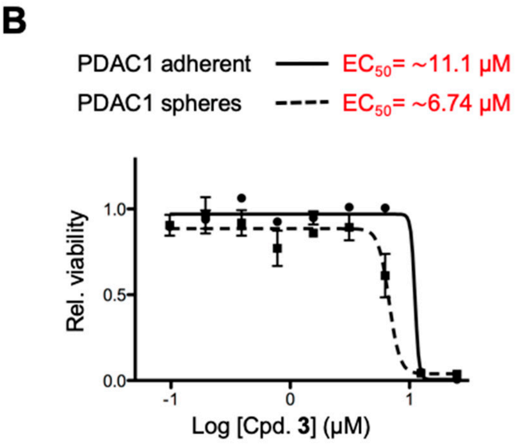

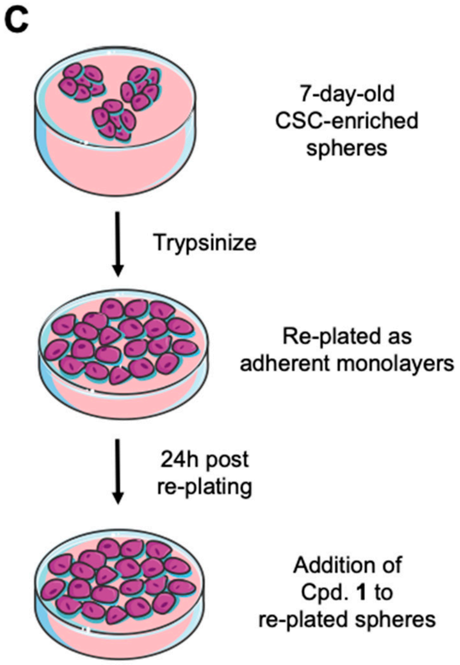

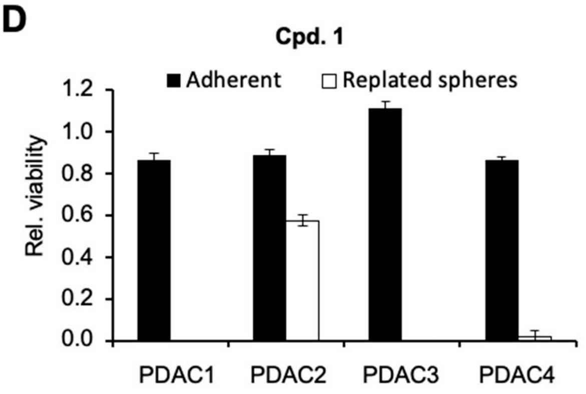

Figure S1. Compounds preferentially target CSCs. (**A**) Top 12 compounds belonging to Series 1 and Series 2 and their respective PI3K and mTOR activity (IC_{50}). (**B**) Viability curves performed on PDAC1 adherent 2D monolayers or 3D spheres treated with increasing concentrations of compound Cpd. **3** for 30 h. Indicated in red are the calculated EC_{50}. (**C**) Schematic representation of re-plated sphere strategy. (**D**) Analysis of relative cell viability ± standard deviation for PDAC1, PDAC2, PDAC3 and PDAC4 adherent 2D monolayers or re-plated spheres following 72 h treatment with 2.5 µM of Cpd. **1**.

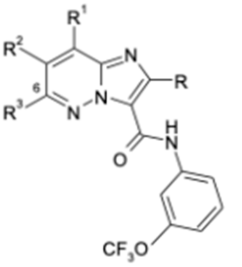

Imidazo[1,2-b]pyridazine scaffold
General Formula for all compounds in table expect for compound **17**

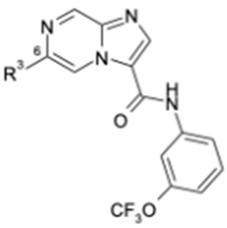

Imidazo[1,2-a]pyrazine scaffold
* General Formula for compound **17**

Cpd	R	R^1	R^2	R^3	EC_{50} (µM)	pKa	cLopP	Basicity
**1**	OMe	H	H	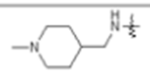	4.64	10.01	4.613	Yes
**2**	H	H	H	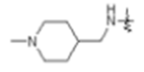	3.89	10.01	4.044	Yes
**13**	H	H	Me	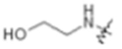	>25	4.42	3.020	No
**14**	H	H	H	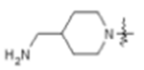	1.42	10.21	3.517	Yes
**15**	H	H	H	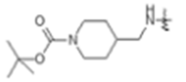	>25	4.86	5.891	No
**16**	H	H	H	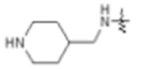	3.26	10.49	3.457	Yes
**17***	-	-	-	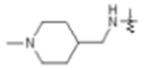	7.22	10.01	4.036	Yes
**18**	H	H	H	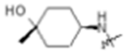	>25	4.99	4.438	No
**19**	H	H	H	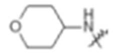	>25	4.36	3.876	No
**20**	H	H	H	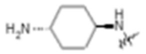	9.02	10.40	3.304	Yes
**21**	H	H	H	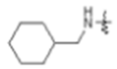	>25	5.2	4.854	No

Figure S2. Cellular activity and calculated physico-chemical properties of compounds from Series 1. Dose-response viability assays on PDAC1 spheres of compounds for 30 h. Calculated EC_{50} is reported in µM. Physico-chemical properties pKa, cLopP calculated with Epik and QikProp respectively from Schrödinger Release 2019-4. Basicity of compounds based on pKa data.

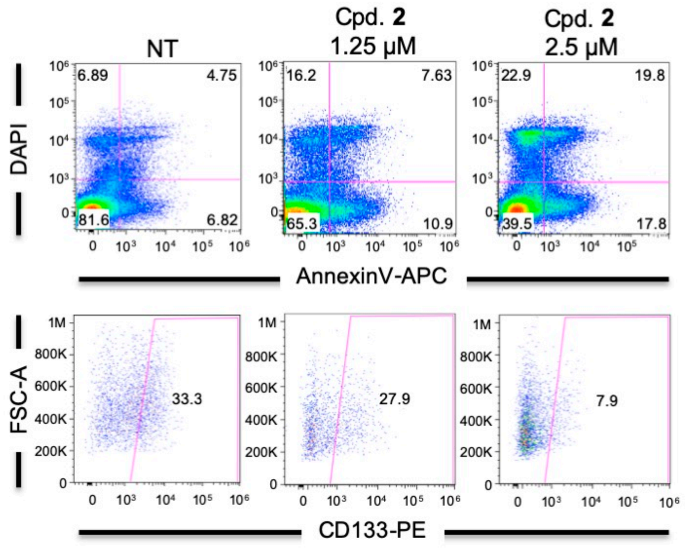

Figure S3. Compound 2 preferentially targets CD133+ CSCs. Top: Representative flow cytometry plots of the percentage of early apoptosis (Annexin-V+), late apoptosis (Annexin-V+/DAPI+) and dead (DAPI+) cells in PDAC2 cells treated with Cpd. **2** at 1.25 or 2.5 μM. Apoptosis was determined 72 h post treatment initiation. Bottom: Representative flow cytometry plots of CD133-PE expression (percentage) in PDAC2 cells 72h following treatment with Cpd. **2** at 1.25 or 2.5 μM.

A

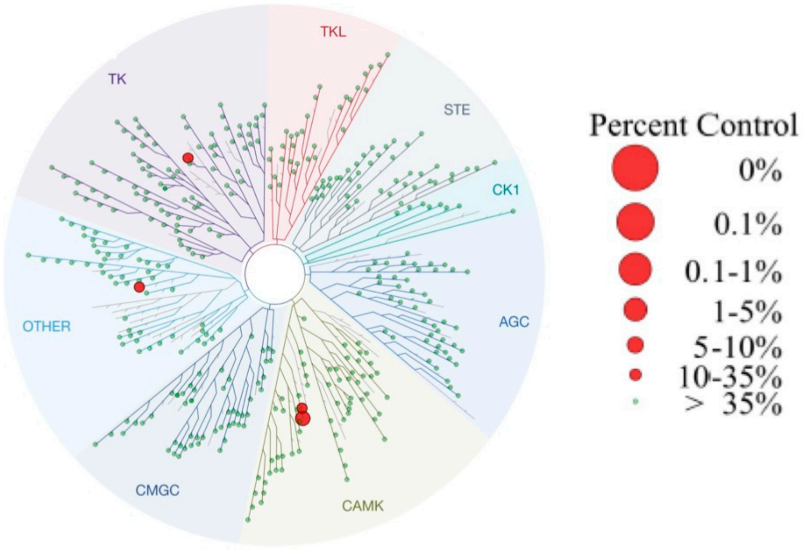

B

	IC$_{50}$ (nM)			
	**PIM1**	**PIM3**	**HASPIN**	**TYK2**
**Cpd 2**	90	497	915	65000
**Cpd 1**	>100000	>100000	>100000	

Figure S4. Selectivity profile of compound 2. (**A**) Kinases found to bind (percent of control < 35) are marked with red circles, where larger circles indicate higher-affinity binding. Image generated using TREE*spot*™ Software Tool and reprinted with permission from KINOME*scan*®, a division of DiscoveRx Corporation, © DISCOVERX CORPORATION 2010. (**B**) IC$_{50}$ (nM) of Cp1s 1 and 2 againsy PIM1, PIM3, HASPIN or TYK2.

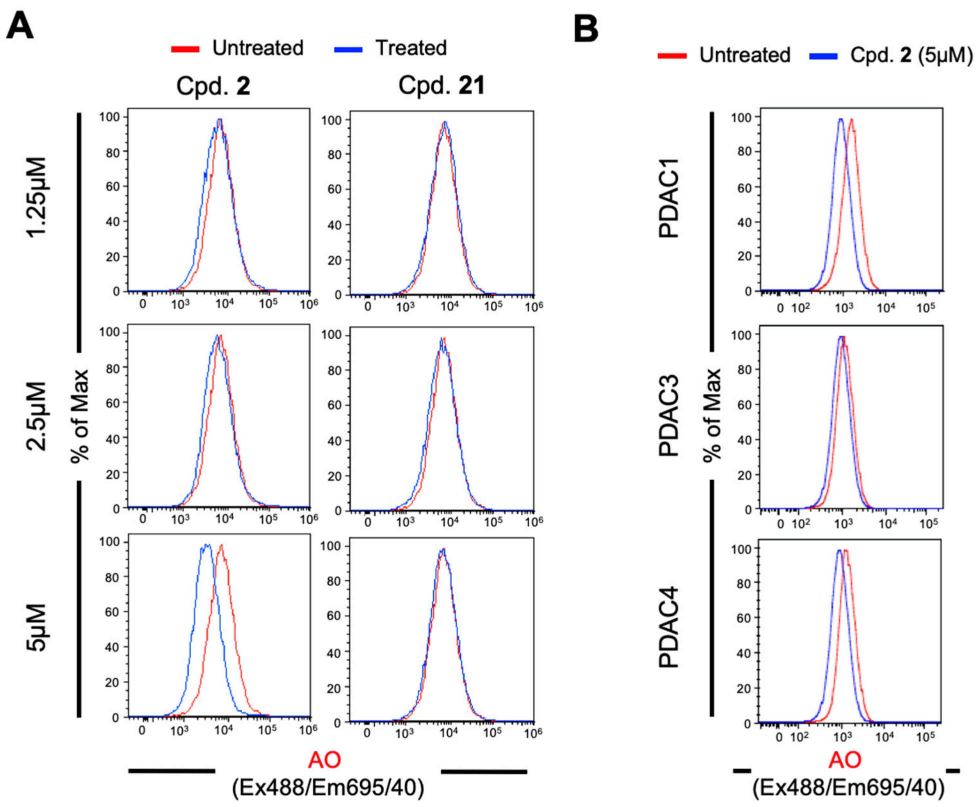

Figure S5. Compound 2 induces lysosomal membrane permeabilization as early as 6 hours and across all PDAC cultures. (**A**) Representative flow cytometric analysis of Acridine Orange in re-plated PDAC2 spheres. Cultures were either non-treated (NT) or treated with 1.25, 2.5 or 5 μM of Cpd. **2** or Cpd. **21**, and fluorescence was assessed at 6 h post treatment. (**B**) Representative flow cytometric analysis of Acridine Orange in re-plated PDAC1, PDAC3 or PDAC4 spheres. Cultures were either non-treated (NT) or treated with 5 μM of Cpd. **2**, and fluorescence was assessed at 24 h post treatment.

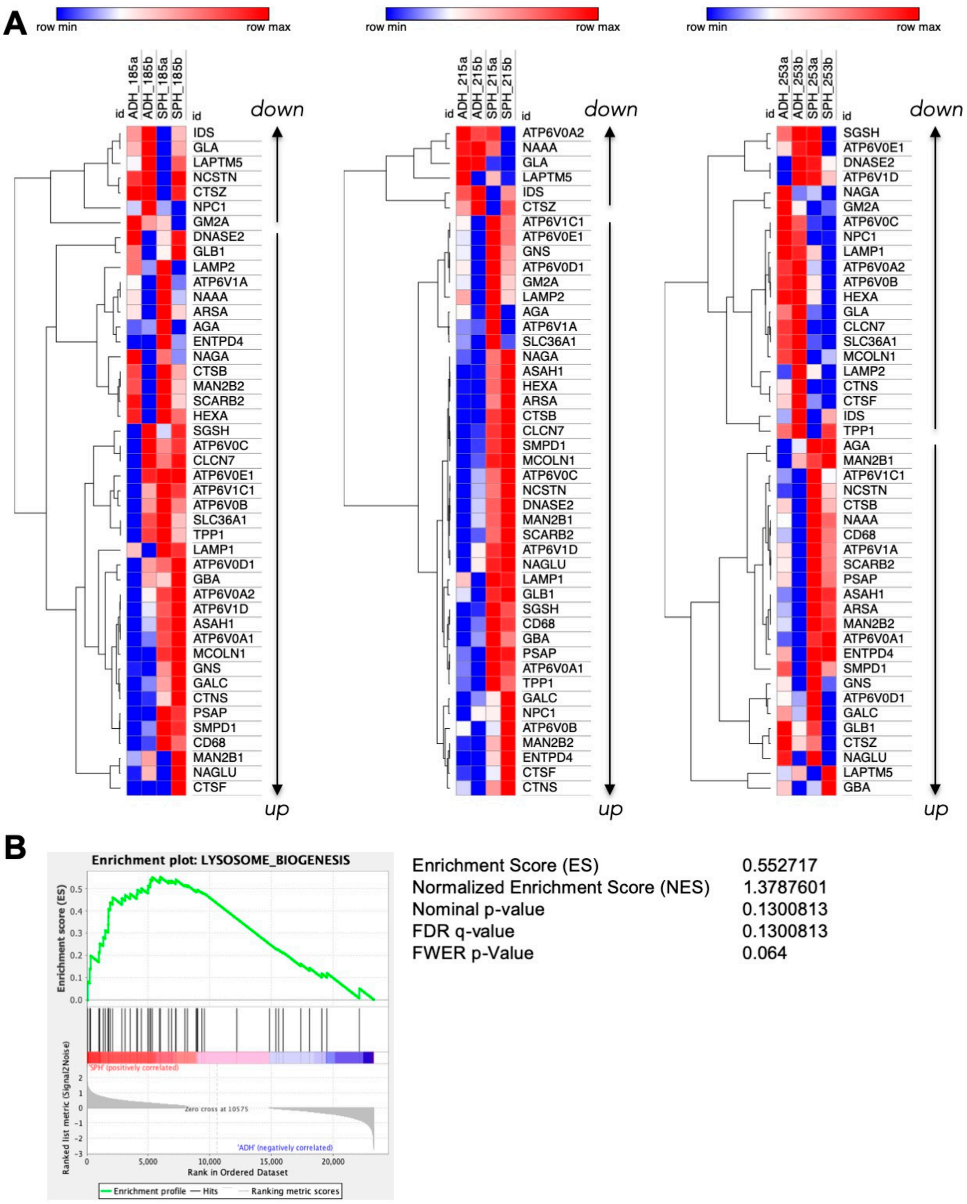

Figure S6. PDAC CSCs upregulate genes involved in lysosome biogenesis. (**A**) Heat maps illustrating expression of lysosome biogenesis genes in three adherent and sphere-derived cultures from 3 PDX-derived cultures (n = 2 samples/condition). Data are derived from the dataset E-MTAB-3808, archived in ArrayExpress. Represented are FPMK values. Samples are clustered hierarchically by rows to visually demonstrate genes that go down and up, as indicated by the arrows. (**B**) Gene set enrichment analysis (GSEA) of Lysosome Biogenesis in adherent (non-CSC) and sphere-derived (CSC) isolated from PDX-derived cultures (n = 2 samples/condition).

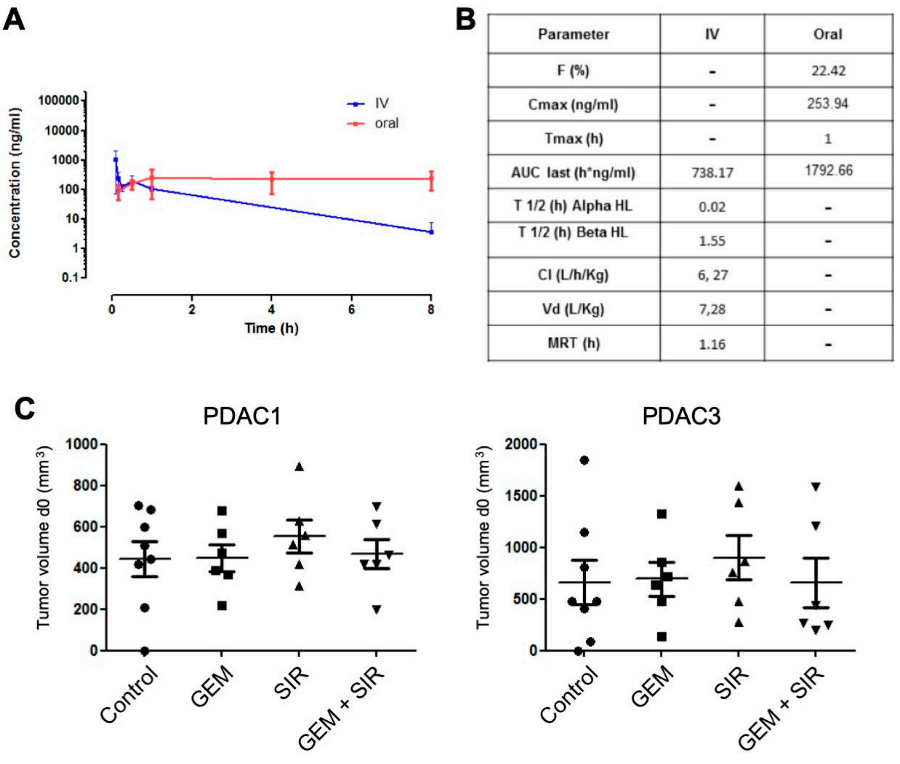

Figure S7. *In vivo* studies. (**A**) Pharmacokinetic study of Cpd. **2**. In the graph is represented the plasmatic concentration (Cp) evolution vs time after IV (5 mg/kg) and oral (100 mg/Kg) administration. In the table are represented the pharmacokinetic parameters. (**B**) PDAC1 and PDAC3 tumor volumes (mm^3) in mice randomized to respective treatment groups. One-way ANOVA analysis indicated no significant differences between randomized treatment groups at the beginning of the treatment.

Table S1. Kinase orthogonal assay results.

DiscoveRx Gene Symbol	% Ctrl	DiscoveRx Gene Symbol	% Ctrl	DiscoveRx Gene Symbol	% Ctrl
AAK1	89	FES	87	PCTK3	100
ABL1(E255K)-P	85	FGFR1	100	PDGFRA	81
ABL1(F317I)-nonP	61	FGFR2	100	PDGFRB	92
ABL1(F317I)-P	94	FGFR3	98	PDPK1	100
ABL1(F317L)-nonP	66	FGFR3(G697C)	99	PFCDPK1(P.falciparum)	88
ABL1(F317L)-P	100	FGFR4	100	PFPK5(P.falciparum)	97
ABL1(H396P)-nonP	93	FGR	77	PFTAIRE2	92
ABL1(H396P)-P	100	FLT1	100	PFTK1	97
ABL1(M351T)-P	100	FLT3	92	PHKG1	100
ABL1(Q252H)-nonP	94	FLT3(D835H)	74	PHKG2	89
ABL1(Q252H)-P	100	FLT3(D835V)	0.45	PIK3C2B	48
ABL1(T315I)-nonP	70	FLT3(D835Y)	65	PIK3C2G	59
ABL1(T315I)-P	100	FLT3(ITD)	100	PIK3CA	100
ABL1(Y253F)-P	100	FLT3(ITD,D835V)	9.1	PIK3CA(C420R)	100
ABL1-nonP	100	FLT3(ITD,F691L)	34	PIK3CA(E542K)	92
ABL1-P	95	FLT3(K663Q)	92	PIK3CA(E545A)	100
ABL2	96	FLT3(N841I)	60	PIK3CA(E545K)	87
ACVR1	99	FLT3(R834Q)	100	PIK3CA(H1047L)	93

ACVR1B	97	FLT3-autoi.	82	PIK3CA(H1047Y)	96
ACVR2A	100	FLT4	100	PIK3CA(I800L)	81
ACVR2B	100	FRK	100	PIK3CA(M1043I)	90
ACVRL1	100	FYN	96	PIK3CA(Q546K)	89
ADCK3	100	GAK	89	PIK3CB	91
ADCK4	99	GCN2(KD2,S808G)	73	PIK3CD	85
AKT1	100	GRK1	86	PIK3CG	100
AKT2	100	GRK2	79	PIK4CB	99
AKT3	100	GRK3	76	PIKFYVE	45
ALK	100	GRK4	93	PIM1	5
ALK(C1156Y)	85	GRK7	41	PIM2	72
ALK(L1196M)	100	GSK3A	100	PIM3	18
AMPK-alpha1	100	GSK3B	97	PIP5K1A	100
AMPK-alpha2	100	HASPIN	30	PIP5K1C	74
ANKK1	94	HCK	96	PIP5K2B	95
ARK5	88	HIPK1	100	PIP5K2C	58
ASK1	100	HIPK2	68	PKAC-alpha	99
ASK2	99	HIPK3	100	PKAC-beta	88
AURKA	96	HIPK4	88	PKMYT1	75
AURKB	97	HPK1	92	PKN1	100
AURKC	95	HUNK	100	PKN2	79
AXL	65	ICK	93	PKNB(M.tuberculosis)	90
BIKE	83	IGF1R	100	PLK1	98
BLK	100	IKK-alpha	97	PLK2	91
BMPR1A	97	IKK-beta	100	PLK3	97
BMPR1B	99	IKK-epsilon	100	PLK4	82
BMPR2	87	INSR	100	PRKCD	80
BMX	100	INSRR	94	PRKCE	70
BRAF	100	IRAK1	100	PRKCH	99
BRAF(V600E)	100	IRAK3	100	PRKCI	85
BRK	99	IRAK4	100	PRKCQ	55
BRSK1	100	ITK	95	PRKD1	98
BRSK2	100	JAK1(JH1D.-cat.)	100	PRKD2	87
BTK	100	JAK1(JH2D.-pseudoK.)	100	PRKD3	92
BUB1	62	JAK2(JH1D.-cat.)	96	PRKG1	100
CAMK1	98	JAK3(JH1D.-cat.)	100	PRKG2	100
CAMK1B	97	JNK1	92	PRKR	83
CAMK1D	94	JNK2	99	PRKX	100
CAMK1G	92	JNK3	100	PRP4	100
CAMK2A	82	KIT	99	PYK2	95
CAMK2B	92	KIT(A829P)	91	QSK	100
CAMK2D	99	KIT(D816H)	91	RAF1	100
CAMK2G	100	KIT(D816V)	89	RET	88
CAMK4	100	KIT(L576P)	88	RET(M918T)	100
CAMKK1	94	KIT(V559D)	95	RET(V804L)	100
CAMKK2	100	KIT(V559D,T670I)	100	RET(V804M)	97
CASK	93	KIT(V559D,V654A)	91	RIOK1	76

CDC2L1	100	KIT-autoi.	100	RIOK2	99
CDC2L2	99	LATS1	100	RIOK3	78
CDC2L5	100	LATS2	99	RIPK1	100
CDK11	97	LCK	77	RIPK2	100
CDK2	100	LIMK1	79	RIPK4	96
CDK3	85	LIMK2	99	RIPK5	84
CDK4	100	LKB1	96	ROCK1	50
CDK4-cyclinD1	100	LOK	90	ROCK2	94
CDK4-cyclinD3	100	LRRK2	68	ROS1	91
CDK5	100	LRRK2(G2019S)	93	RPS6KA4(KD1-N-t.)	92
CDK7	70	LTK	93	RPS6KA4(KD2-C-t.)	100
CDK8	58	LYN	98	RPS6KA5(KD1-N-t.)	100
CDK9	98	LZK	92	RPS6KA5(KD2-C-t.)	92
CDKL1	97	MAK	94	RSK1(KD1-N-t.)	97
CDKL2	98	MAP3K1	100	RSK1(KD2-C-t.)	89
CDKL3	91	MAP3K15	100	RSK2(KD1-N-t.)	89
CDKL5	100	MAP3K2	92	RSK2(KD2-C-t.)	100
CHEK1	86	MAP3K3	96	RSK3(KD1-N-t.)	93
CHEK2	95	MAP3K4	86	RSK3(KD2-C-t.)	74
CIT	60	MAP4K2	74	RSK4(KD1-N-t.)	92
CLK1	100	MAP4K3	95	RSK4(KD2-C-t.)	84
CLK2	81	MAP4K4	89	S6K1	93
CLK3	93	MAP4K5	92	SBK1	100
CLK4	100	MAPKAPK2	94	SGK	85
CSF1R	100	MAPKAPK5	100	SgK110	72
CSF1R-autoi.	88	MARK1	100	SGK2	100
CSK	100	MARK2	96	SGK3	100
CSNK1A1	100	MARK3	100	SIK	99
CSNK1A1L	95	MARK4	100	SIK2	97
CSNK1D	100	MAST1	59	SLK	79
CSNK1E	95	MEK1	95	SNARK	89
CSNK1G1	99	MEK2	90	SNRK	98
CSNK1G2	100	MEK3	94	SRC	99
CSNK1G3	100	MEK4	100	SRMS	87
CSNK2A1	98	MEK5	73	SRPK1	95
CSNK2A2	39	MEK6	98	SRPK2	94
CTK	70	MELK	100	SRPK3	77
DAPK1	82	MERTK	88	STK16	96
DAPK2	89	MET	100	STK33	100
DAPK3	75	MET(M1250T)	100	STK35	92
DCAMKL1	85	MET(Y1235D)	93	STK36	100
DCAMKL2	97	MINK	94	STK39	99
DCAMKL3	100	MKK7	100	SYK	94
DDR1	92	MKNK1	86	TAK1	70
DDR2	89	MKNK2	96	TAOK1	82
DLK	100	MLCK	100	TAOK2	100
DMPK	88	MLK1	95	TAOK3	99

DMPK2	97	MLK2	100	TBK1	100
DRAK1	97	MLK3	100	TEC	96
DRAK2	80	MRCKA	100	TESK1	90
DYRK1A	100	MRCKB	100	TGFBR1	100
DYRK1B	92	MST1	100	TGFBR2	100
DYRK2	86	MST1R	100	TIE1	90
EGFR	94	MST2	100	TIE2	100
EGFR(E746-A750del)	77	MST3	96	TLK1	98
EGFR(G719C)	82	MST4	100	TLK2	100
EGFR(G719S)	100	MTOR	100	TNIK	81
EGFR(L747-E749del, A750P)	84	MUSK	99	TNK1	56
EGFR(L747-S752del, P753S)	92	MYLK	100	TNK2	99
EGFR(L747-T751del,Sins)	87	MYLK2	97	TNNI3K	100
EGFR(L858R)	94	MYLK4	100	TRKA	82
EGFR(L858R,T790M)	100	MYO3A	91	TRKB	89
EGFR(L861Q)	94	MYO3B	100	TRKC	70
EGFR(S752-I759del)	97	NDR1	89	TRPM6	100
EGFR(T790M)	96	NDR2	91	TSSK1B	95
EIF2AK1	95	NEK1	99	TSSK3	95
EPHA1	100	NEK10	85	TTK	63
EPHA2	100	NEK11	79	TXK	100
EPHA3	92	NEK2	94	TYK2(JH1D.-cat.)	65
EPHA4	88	NEK3	100	TYK2(JH2D.-pseudoK.)	12
EPHA5	100	NEK4	100	TYRO3	89
EPHA6	86	NEK5	98	ULK1	98
EPHA7	100	NEK6	100	ULK2	100
EPHA8	100	NEK7	92	ULK3	78
EPHB1	97	NEK9	88	VEGFR2	100
EPHB2	95	NIK	100	VPS34	75
EPHB3	86	NIM1	78	VRK2	100
EPHB4	93	NLK	97	WEE1	96
EPHB6	92	OSR1	100	WEE2	92
ERBB2	88	p38-alpha	96	WNK1	100
ERBB3	100	p38-beta	100	WNK2	77
ERBB4	100	p38-delta	100	WNK3	99
ERK1	100	p38-gamma	91	WNK4	100
ERK2	100	PAK1	100	YANK1	100
ERK3	100	PAK2	94	YANK2	100
ERK4	100	PAK3	98	YANK3	99
ERK5	100	PAK4	100	YES	95
ERK8	96	PAK6	98	YSK1	95
ERN1	79	PAK7	90	YSK4	71
FAK	100	PCTK1	100	ZAK	96
FER	98	PCTK2	100	ZAP70	95
